# Spherical Amides with *C*_3_ Symmetry: Improved Synthetic Approach and Structural/Optical Analysis

**DOI:** 10.3390/molecules30051074

**Published:** 2025-02-26

**Authors:** Daiki Koike, Hyuma Masu, Haruka Uno, Shoko Kikkawa, Hidemasa Hikawa, Isao Azumaya

**Affiliations:** 1 Faculty of Pharmaceutical Sciences, Toho University, 2-2-1 Miyama, Funabashi 274-8510, Chiba, Japan; 2 Center for Analytical Instrumentation, Chiba University, 1-33 Yayoi-cho, Inage-ku, Chiba 263-8522, Japan

**Keywords:** chiral macrocycle, organic cage, cyclic amide, molecular scaffold

## Abstract

A spherical amide with *C*_3_ symmetry was synthesized by a one-step cyclization reaction using triphenylphosphine and hexachloroethane as coupling reagents. This method enabled synthesis of *N*-benzyl and *N*-allyl derivatives, which could not be obtained by the previously reported method. The optical resolution of each compound was measured, and their electronic circular dichroism spectra revealed that they were mirror images. The high structural symmetry resulted in a higher Δε (molar absorption difference against right or left circular polarization: *ε_L_* − *ε_R_* value compared to that of another structural isomer synthesized previously. The absolute structure of the enantiopure crystal of the *N*-benzyl derivative was determined using the Flack parameter obtained by X-ray crystallographic analysis.

## 1. Introduction

Macrocyclic compounds such as calixarenes [1,2,3,4], cyclophanes [5,6,7,8], and porphyrins [9,10,11] have attracted much attention as important building blocks in materials science, supramolecular chemistry, and chemical biology. Among these, chiral macrocycles have potential applications in enantioselective recognition [12,13] or as asymmetric catalysts [14,15,16] or circularly polarized luminescence (CPL) emitters [17,18]. Three-dimensional molecules with internal cavities such as fullerenes [19,20,21,22], carbon nanotubes [23,24,25], and organic cages [26,27,28,29] are useful in a wide range of fields. However, both these types of molecules are difficult to synthesize and/or functionalize. Exploring new types of such molecules is essential to overcome these challenges and broaden their applications.

**Figure 1:**
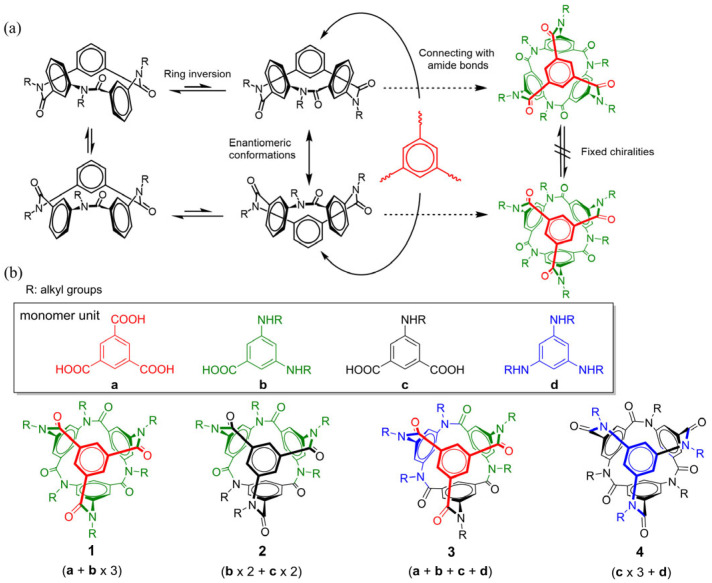
(**a**) Equilibrium of racemization of *m*-calix[3]amide. When *m*-calix[3]amide and a benzene ring are connected through amide bonds, spherical molecule **1**, in which the racemization is suppressed, is obtained. (**b**) Four structural isomers (**1**–**4**) of the spherical amide. Each component is colored as follows. a: Red: trimesic acid; b: green: 3,5-bis(alkylamino)benzoic acid; c: black: 3-(alkylamino)isophthalic acid; d: blue: 1,3,5-tri(alkylamino)benzene. R: alkyl group.

We have previously synthesized various macrocycles [30,31,32,33] by exploiting the *cis*-type conformational preference of tertiary aromatic amides [34,35]. Among these macrocycles, *m*-calix[3]amide [36], which is a cyclic trimer consisting of benzamide units, has an axial chirality derived from the direction of the amide bond. However, *m*-calix[3]amide racemizes easily via the inversion of the benzene rings (Figure 1a). Introducing bulky substituents can suppress such racemization by increasing the energy barrier for benzene ring inversion, but the choice of substituents is limited. To overcome this problem, we designed a new type of molecule that contains an additional benzene unit that bridges the *m*-calix[3]amide framework to fix its chirality. It consists of four benzene rings connected at all *meta* positions of the *m*-calix[3]amide framework by six tertiary amide bonds, resulting in a spherical structure, which we term “spherical amide”. The skeletons of these molecules have fixed axial chirality and feature six amide nitrogen atoms that are amenable to further functionalization. This class of molecule has four structural isomers, distinguished by the directional combination of the amide bonds arising from the different monomers (Figure 1b, compound **1**–**4**).

**Figure 2:**
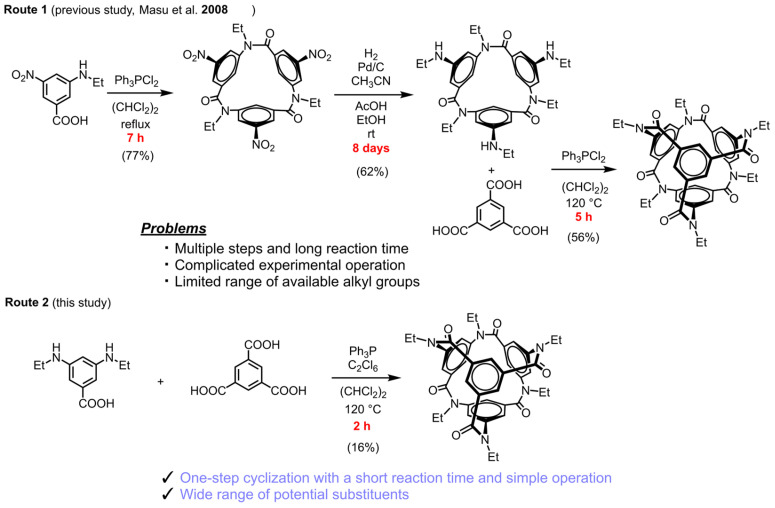
Synthetic routes to spherical amide **1** [37].

In our previous study [37], we synthesized isomer **1** via a two-step amide condensation process (Figure 2, route 1). The first step involved cyclization using 3-(ethylamino)-5-nitrobenzoic acid as a monomer, and the second step entailed condensation of the cyclic trimer and trimesic acid. In both steps, dichlorotriphenylphosphorane (Ph_3_PCl_2_) was employed as the coupling reagent [38]. In addition, we used the mono-*N*-alkylation reaction starting from the nitro group, as developed by Sajiki et al. [39], to decrease the number of reaction steps. This approach simultaneously reduced the three nitro groups on the cyclic trimer and introduced alkyl groups. The overall yield was 18%, which was acceptable; however, this method had several drawbacks. First, the mono-*N*-alkylation reaction took 8 days. Second, the process involved complicated operations such as frequently replacing molecular sieves to eliminate NH_3_. Furthermore, the alkyl groups that could be introduced onto amino groups were restricted because the reaction required a nitrile compound as an *N*-alkylating reagent, and some nitriles, such as benzonitrile, were not suitable because they were smoothly reduced under catalytic hydrogenation conditions using a palladium/carbon catalyst. Additionally, for certain alkyl groups, such as the allyl group, the corresponding nitrile compounds were not available. To address these problems, we aimed to develop a new synthetic approach involving one-step cyclization starting from a monomer that had been *N*-alkylated in advance (Figure 2, route 2). This *N*-alkylated monomer had previously been used to synthesize the structural isomer **2** [40]. In this study, we synthesize three derivatives of isomer **1** (**1a**: *N*-ethyl; **1b**: *N*-benzyl; **1c**: *N*-allyl) and analyze their structural and chiroptical properties using nuclear magnetic resonance (NMR) spectroscopy, electronic circular dichroism (ECD), and X-ray crystallography. The effect of molecular symmetry on chiroptical properties is assessed by comparing results for isomer **1** and **2**.

## 2. Results and Discussion

### 2.1. Synthesis of Spherical Amides

**Scheme 1:**
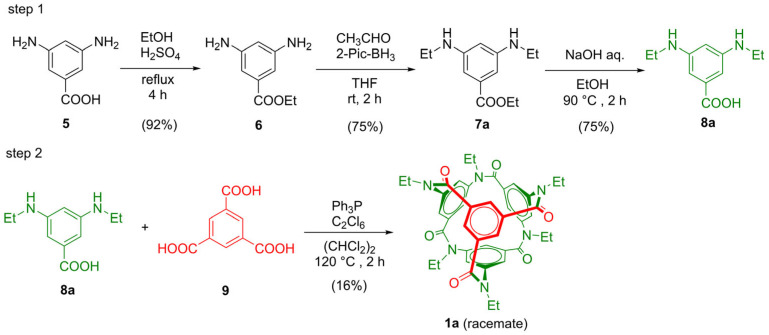
Synthesis of isomer **1** with ethyl groups (**1a**).

First, we synthesized isomer **1a**, which had an ethyl group on each of the six amide nitrogen atoms and was obtained by stepwise condensation in our previous study [37]. Compound **8a** was synthesized as the monomer for three of four subunits of spherical amide **1a** by step 1 in Scheme 1. Ethyl 3,5-diaminobenzoate (**6**), which was synthesized from 3,5-diaminobenzoic acid (**5**) as a starting material by esterification, was *N*-alkylated by reductive amination with 2-picoline borane [41], followed by hydrolysis using aqueous sodium hydroxide (NaOH, 4 M) to afford 3,5-bis(ethylamino)benzoic acid (**8a**). The cyclization condensation reaction of a 3:1 mixture of **8a** and **9** was performed at 120 °C for 2 h in 1,1,2,2-tetrachloroethane using triphenylphosphine (Ph_3_P) and hexachloroethane (C_2_Cl_6_) as coupling reagents (Scheme 1, step 2) [42,43]. The obtained crude product was purified by silica gel column chromatography and gel permeation chromatography (GPC). In this purification, triphenylphosphine oxide and polymeric compounds could be separated as by-products, giving the desired product as a racemic mixture in 16.4% yield. The yields in the previous method were 77%, 62%, and 56% for cyclization, mono-*N*-alkylation, and condensation between cyclic trimer and trimesic acid, respectively, and the overall yield from the monomer was 27% and took over 10 days. By contrast, the reaction time of the one-step cyclization was just 2 h and the experimental operation was simple compared to that in the previous method, although the total yield was slightly lower. The coupling reaction was performed using Ph_3_P and C_2_Cl_6_ to generate Ph_3_PCl_2_ in situ, instead of Ph_3_PCl_2_ itself, because Ph_3_PCl_2_ is highly water sensitive.

**Scheme 2:**
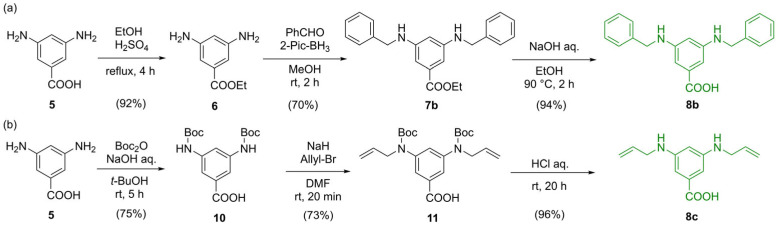
Synthesis of monomer (**a**) **8b**: *N*-benzyl and (**b**) **8c**: *N*-allyl.

**Scheme 3:**
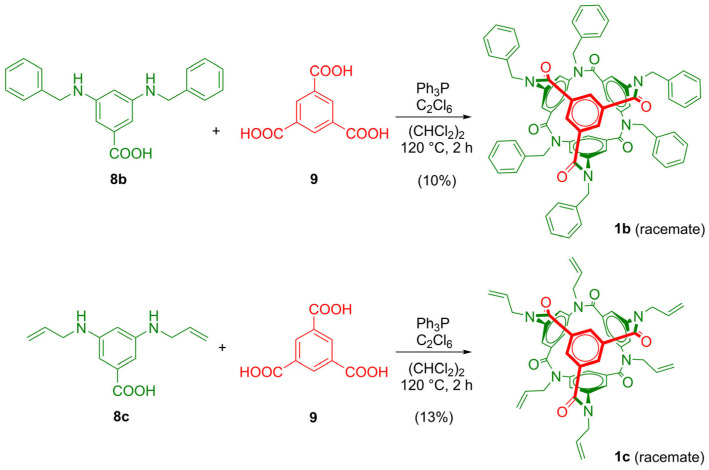
Synthesis of spherical amide **1b** and **1c**.

Next, we attempted to synthesize spherical amides **1b** and **1c** with benzyl and allyl groups on the six amide nitrogen atoms, respectively. Benzyl groups were chosen for their electronic effects and their ability as protecting groups, which could either impart chiroptical properties to the spherical amides or transform the tertiary amide bonds into secondary ones. Allyl groups were selected because of their reactivity and potential for further functionalization, which could expand the structural diversity of spherical amides. However, **1b** and **1c** could not be synthesized using the previous method because of the limitations of the nitrile reagents, i.e., benzonitrile was smoothly reduced and no suitable nitrile compound was available. Thus, we employed route 2, which involves *N*-alkylation in advance followed by one-step cyclization, to synthesize **1b** and **1c**. Monomer **8b** was synthesized in a manner analogous to the synthesis of **8a** using benzaldehyde (Scheme 2a). Similarly, as shown in Scheme 2b, compound **5** was protected with a Boc group, *N*-alkylated using sodium hydride and allyl bromide, and subsequently deprotected to give monomer **8c**. The cyclization reaction was performed according to Scheme 3 using monomer **8b** and **8c**. After purification, spherical amide **1b** was obtained in 10.3% yield. Spherical amide **1c**, which has acid-labile allyl groups, was obtained in 12.7% yield even though HCl was generated during the cyclization reaction. Argon bubbling was used to remove the generated HCl from the reaction solution.

### 2.2. NMR Spectroscopic Analysis

**Figure 3:**
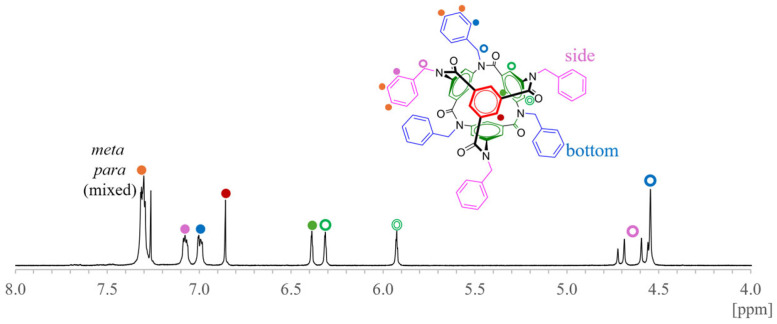
^1^H NMR spectrum of **1b**. The benzene or benzyl units in the structure are indicated as follows. Red: trimesic acid (core); green: 3,5-diamino benzoic acid (core); pink: *N*-benzyl groups at the side; blue: *N*-benzyl groups at the bottom.

The ^1^H NMR spectrum of **1b** is shown in Figure 3. All signals were assigned using ^1^H, ^13^C, ^1^H-^1^H COSY, ^1^H-^13^C HMBC, ^1^H-^13^C HSQC, and ^1^H-^1^H NOESY NMR spectra (S1 and S2 in Supplementary Material). The integration of each peak corresponded to the expected number of protons for the structure of the spherical amide, further supporting the assigned structure. In the aromatic region, the signals corresponding to protons on the benzene rings of the spherical core structure appeared at higher magnetic field than those of the monomers. This suggests that the molecule has a cyclic structure because the protons on each benzene ring are influenced by anisotropic effects from neighboring benzene rings after cyclization. Additionally, the signals of **1b** were sharp, indicating the core structure is rigidly fixed. By contrast, the signals of the cyclic trimer were generally broad at room temperature because of the inversion of the benzene rings. In the low magnetic field region, mixed signals corresponding to protons on the *para* and *meta* positions of the six benzene rings in the side chains were observed. The protons at *ortho* and benzyl positions appeared as two sets of signals at 7.12–6.95 and 4.75–4.50 ppm, respectively. All sets of signals appeared to be equivalent because of their symmetry. These results indicated that **1b** had a sphere-shaped core structure with *C*_3_ symmetry, and the side chains can be classified as bottom and side relative to the cyclic core, as illustrated in Figure 3.

### 2.3. Optical Resolution and ECD Spectral Analysis

**Figure 4:**
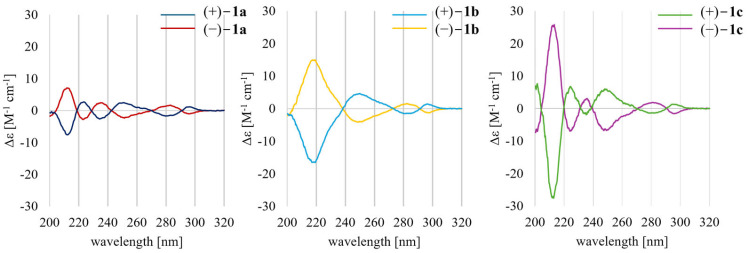
ECD spectra of **1a** (*N*-ethyl), **1b** (*N*-benzyl), and **1c** (*N*-allyl) in acetonitrile solution. The sign of (+) or (−) was determined by optical rotation at 589 nm in acetonitrile solution.

Most molecules with axial chirality racemize over time or at elevated temperature. By contrast, the spherical amides do not racemize because of their covalently fixed axial chirality. For the racemic mixtures of **1a**, **1b**, and **1c**, optical resolution was achieved by chiral HPLC method with chiral stationary phase, resulting in two enantiopure components in a 1:1 ratio (for details, see SI). The enantiomeric purity of each compound was confirmed to be 100% ee. The ECD spectra of these enantiomers in acetonitrile solution are shown in Figure 4. The spectra exhibited a mirror-image Cotton effect. At longer wavelengths, the Cotton effects of all derivatives were nearly identical. Specifically, the Δε values of the first positive Cotton effect of (−)-**1a**, (−)-**1b**, and (−)-**1c** at the longest wavelength region were 1.7, 1.6, and 1.8, respectively. These results suggest that the peaks originate from the core structure and are minimally influenced by the substituents because of the rigidity of the spherical amide skeleton. At shorter wavelengths, the Cotton effects increased markedly because of the ultraviolet (UV) absorption of benzyl or allyl groups. For example, around 210–220 nm, Δε values of (−)-**1a**, (−)-**1b**, and (−)-**1c** were 7.1 (212 nm), 16.4 (219 nm), and 25.6 (212 nm), respectively.

**Figure 5:**
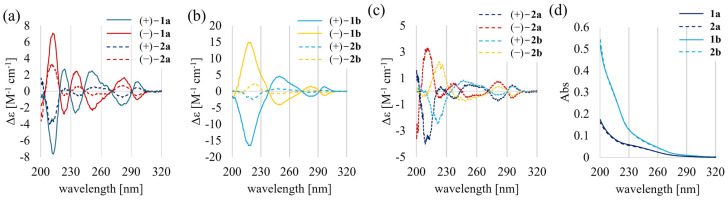
Comparison of ECD spectra of (**a**) **1a** and **2a** (*N*-ethyl), (**b**) **1b** and **2b** (*N*-benzyl), and (**c**) **2a** and **2b** in acetonitrile solution. (**d**) UV absorption spectra of the same compounds in acetonitrile solution.

Next, we examined the differences in chiroptical properties between two structural isomers with different symmetries using UV and ECD spectroscopy. In the ECD spectra of both isomers (Figure 5a,b), the Cotton effects of isomer **1** were stronger than those of isomer **2** (Figure 1) across all wavelengths. Meanwhile, the UV spectra of the two structural isomers showed no obvious differences (Figure 5d). Interestingly, when comparing the ECD spectra of isomer **2** with ethyl and benzyl groups (Figure 5c), the Cotton effects of **2b** (*N*-benzyl) were weaker than those of **2a** (*N*-ethyl), which is the opposite trend to that observed for isomer **1**. The Cotton effects of isomer **2** may be weakened by dipole-moment cancelation arising from its asymmetric structure. By contrast, the *C*_3_-symmetric structure of isomer **1** produced stronger ECD signals, suggesting its potential as an efficient chiroptical material, such as a CPL emitter, if fluorescent substituents with absorption maxima in the longer wavelength region are introduced.

### 2.4. X-Ray Crystallographic Analysis

**Figure 6:**
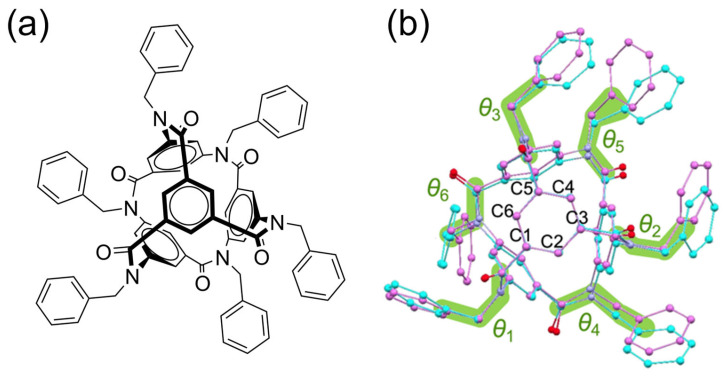
Crystal structures of chiral (+)-1b. (**a**) Molecular structure. (**b**) Superimposed structures. Pink: molecule A with benzene rings numbered C1–C6; pale blue: molecule B with benzene rings numbered C101–C106.

Next, a crystal of (+)-**1b** was obtained by slow evaporation from the solution used for optical resolution (**1b** in *n*-hexane–CH_2_Cl_2_ = 1:1 with 1% 2-propanol), and X-ray crystallographic analysis was performed (for details, see SI [S1, S2]). The crystal belonged to the space group *P*2_1_, and the molecule had an expected spherical shape (Figure 6). The Flack parameter of this crystal was 0.018(18) [44,45]; therefore, the absolute structure was identified as the structure shown in Figure 6a. The crystal consisted of two independent molecular conformations (molecule A and B) per asymmetric unit with solvent molecules (CH_2_Cl_2_ and H_2_O). The individual structures possessed almost identical spherical core structures. Furthermore, the conformations of benzyl groups were only slightly different (for details, see below).

**Figure 7:**
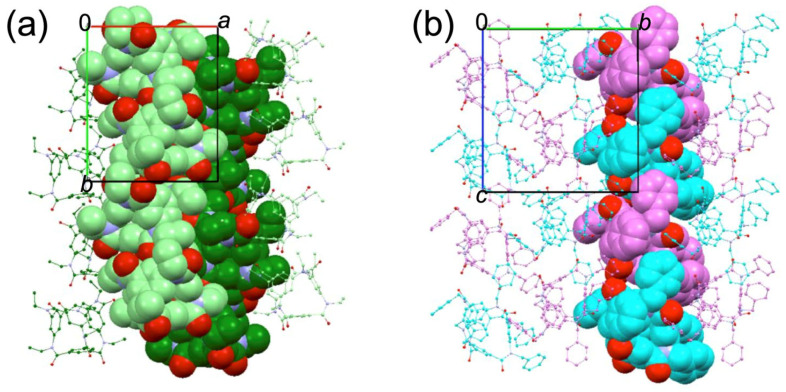
Molecular arrangements in the crystals of (+)-1a (R = Et, CCDC code: LOJMIH) and (+)-1b (R = Bn). (**a**) Crystal structures of (+)-1a. The view is along the *a* axis. Green: molecule C; pale green: molecule D. (**b**) Crystal structures of (+)-1b. The view is along the *c* axis. Pink: molecule A; pale blue: molecule B.

Next, we compared the crystal structure of (+)-**1b** with that of (+)-**1a** (*N*-ethyl derivative), which was obtained in a previous study (CCDC code: LOJMIH [37]), to further investigate the structural properties of spherical amides. The internal cavity of the core structure of (+)-**1b** was too narrow to include a guest molecule, as was that of (+)-**1a**. Both compounds crystallized in the Sohncke space group *P*2_1_ with two independent molecular conformations in the asymmetric unit (Z’ = 2). However, the arrangements of molecules were different. The crystal structure of (+)-**1a** (consisting of molecule C and D) contained two types of one-dimensional (1D) columnar structures composed of molecules of the same conformation (…C…C… and …D…D…), which were both oriented along the *b*-axis. By contrast, the crystal of (+)-**1b** possessed a 1D columnar structure consisting of alternating molecules A and B along the *c* axis (…A…B…A…B…) (Figure 7).

In both crystal structures, although the 1D columnar structures were constructed via C=O…H-C (sp^2^) and C=O…O (H_2_O) interactions, these interactions were also observed between columnar structures; i.e., no characteristic interactions specific to the columnar structures were observed. For both crystals, no channel structure was formed. The direction of substituents relative to the spherical skeleton differed markedly between (+)-**1a** and (+)-**1b**. In the crystal of (+)-**1b**, the conformations of the benzyl groups of molecule A and B were consistent and showed similar dihedral angles *θ* of their C(=O)-N-C(methylene)-C(benzene) bonds (Figure 6b, Table 1 and Table S1). By contrast, in the crystal of (+)-**1a**, the directions of the ethyl groups in molecule C and D differed somewhat (Table S1).

## 3. Materials and Methods

### 3.1. General

All of the starting materials and solvents were purchased from KANTO CHEMICAL Co., Inc. (Tokyo, Japan), TCI (Tokyo, Japan), FUJIFILM Wako (Osaka, Japan), JUNSEI CHEMICAL CO., Ltd. (Tokyo, Japan), Sigma-Aldrich Co. LLC (St. Louis, MO, USA), NACALAI TESQUE, Inc. (Kyoto, Japan). Ethyl 3,5-diaminobenzoate was synthesized following the known procedure [46]. ^1^H, ^13^C, and all 2D NMR spectra were recorded on JEOL (Tokyo, Japan) JNM-ECS400 spectrometer (400 MHz for ^1^H, 100 MHz for ^13^C), and the internal standards of ^1^H and ^13^C NMR spectra were tetramethylsilane (0.00 ppm) and solvent residual peaks. The following abbreviations are used: s = singlet; d = doublet; t = triplet; q = quartet; dd = double doublet; m = multiplet. Infrared (IR) spectra were recorded on a JASCO (Tokyo, Japan) FT/IR-4100 spectrophotometer. Fast atom bombardment mass spectra (FAB-MS) were performed on a JEOL JMS700 MStation mass system. Atmospheric pressure chemical ionization mass spectra (APCI-MS) were performed on a Thermo Fisher Scientific (Waltham, MA, USA) Exactive. Column chromatography was performed on silica gel (Silica gel PSQ 100B, spherical, 3–100 nm, Fuji Silysia, Aichi, Japan) with a specified solvent. Purifications with preparative GPC were carried out on a Japan analytical industry (Tokyo, Japan) LC-9210II NEXT system using tandem JAIGEL 2HH columns (CHCl_3_ as an eluent, flow rate = 5.0 mL/min) equipped with an ultraviolet (UV) detector monitored at 254 nm. The chiral high performance liquid chromatography (HPLC) analysis was performed on a JASCO PU-2080 liquid chromatograph equipped with a UV detector (JASCO UV-2075) using a CHIRALPAK column (Daicel Chemical Industries, Ltd., Osaka, Japan) (1.0 cm (i.d.) × 25 cm). The UV spectra of ~1.0 × 10^−6^ M solutions in acetonitrile were measured in 10 mm quartz cells on a JASCO V-730 spectrophotometer. The electronic circular dichroism (ECD) spectra of ~1.0 × 10^−5^ M solutions in acetonitrile were measured in 10 mm quartz cells on a JASCO J-1500 spectrophotometer. 

### 3.2. Synthesis

Ethyl 3,5-bis(benzylamino)benzoate (**7b**)

2-picolineborane (10 mmol, 1.1 g, 1.0 eq.) was added to a solution of ethyl 3,5-diaminobenzoate (compound **6**, 10 mmol, 1.8 g) and benzaldehyde (20 mmol, 2.1 g, 2.0 eq.) in MeOH (50 mL), and the mixture was stirred at room temperature for 2 h. The crude product was purified by silica gel column chromatography (ethyl acetate–*n*-hexane = 1:5), and compound **7b** was obtained as a white-yellow solid (2.7 g, 70%); M.p.: 77–79 °C; ^1^H NMR (400 MHz, 298 K, CDCl_3_) δ 7.35–7.23 (m, 10H), 6.75 (d, *J* = 2.1 Hz, 2H), 6.03 (t, *J* = 2.1 Hz, 1H), 4.36–4.24 (m, 6H), 4.04 (s, 2H), 1.35 (t, *J* = 7.1 Hz, 3H); ^13^C NMR (100 MHz, 298 K, CDCl_3_) δ 167.4, 149.3, 139.3, 132.2, 128.7, 127.7, 127.4, 104.2, 101.0, 60.8, 48.4, 14.4; IR (KBr): 2284, 1710, 1598, 1456, 1437, 1361, 1336, 1307, 1240, 1218, 1098, 1025, 770, 749, 732, 698 cm^−1^; HRMS (FAB) *m*/*z* [M+H]^+^ calcd. for C_23_H_25_N_2_O_2_: 361.1916, found: 361.1916.

3,5-Bis(benzylamino)benzoic acid (**8b**)

4 M NaOH was added to a solution of compound **7b** (5 mmol, 1.7 g) in EtOH, and the solution was stirred at 90 °C for 2 h under argon atmosphere. After the reaction was completed, 2 M HCl was added under cooling to make the solution acidic. The mixture was extracted with ethyl acetate, and the organic layer was washed with brine, dried over Na_2_SO_4_, and concentrated in vacuo. The crude product was purified by trituration (toluene–*n*-hexane = 1:1), and compound **8b** was obtained as a brown solid (1.6 g, 94%); M.p.: 128–132 °C; ^1^H NMR (400 MHz, 298 K, DMSO-*d*_6_) δ 12.29 (s, 1H), 7.33–7.26 (m, 8H), 7.25–7.17 (m, 2H), 6.45 (d, *J* = 2.1 Hz, 2H), 6.18 (t, *J* = 5.7 Hz, 2H), 6.03 (t, *J* = 2.1 Hz, 1H), 4.17 (d, *J* = 5.7 Hz, 4H); ^13^C NMR (100 MHz, 298 K, DMSO-*d*_6_) δ 171.9, 149.1, 141.5, 140.8, 128.7, 127.7, 126.9, 103.9, 98.5, 47.3; IR (KBr): 3431, 3026, 2856, 2647, 2527, 1687, 1611, 1494, 1455, 1433, 1403, 1379, 1351, 1306, 1288, 1261, 1222, 931, 830, 764, 742, 726, 703, 695, 674 cm^−1^; HRMS (FAB) *m*/*z* [M+H]^+^ calcd. for C_21_H_21_N_2_O_2_: 333.1603, found: 333.1603.

3,5-Bis[(*tert*-butoxycarbonyl)amino]benzoic acid (**10**)

Boc_2_O (65 mmol, 14.2 g, 2.2 eq.) was added to a solution of 3,5-diaminobenzoic acid (compound **5**, 30 mmol, 4.6 g) in *tert*-butyl alcohol (35 mL) and 1 M NaOH (35 mL), and the rection mixture was stirred at room temperature for 5 h. After the reaction was completed, the mixture was washed with cyclohexane three times, and 1 M HCl was added under cooling to make the solution acidic. The mixture was extracted with ethyl acetate three times, and the organic layer was washed with brine three times, dried over Na_2_SO_4_, and concentrated in vacuo. The residue was purified by trituration with *n*-hexane to obtain compound **10** as brown solid (7.9 g, 75%); M.p.: 185–191 °C; ^1^H NMR (400 MHz, 298 K, DMSO-*d*_6_) δ 9.50 (s, 2H), 7.87 (t, *J* = 1.8 Hz, 1H), 7.70 (d, *J* = 1.8 Hz, 2H), 1.48 (s, 18H); ^13^C NMR (100 MHz, 298 K, DMSO-*d*_6_) δ 167.8, 153.2, 140.5, 131.9, 113.7, 112.7, 79.7, 28.6; IR (KBr): 3349, 2982, 1703, 1613, 1548, 1427, 1391, 1252, 1160, 863, 692 cm^−1^; HRMS (FAB) *m*/*z* [M+Na]^+^ calcd. for C_17_H_24_N_2_O_6_Na: 375.1531, found: 375.1532.

3,5-Bis[allyl(*tert*-butoxycarbonyl)amino]benzoic acid (**11**)

Sodium hydride (60%, dispersion in paraffin liquid) (40 mmol, 1.6 g, 4.0 eq.) was added to a solution of compound **10** (10 mmol, 3.5 g) in DMF anhydrous (50 mL), and the reaction mixture was stirred at room temperature for 1.5 h. Then, allyl bromide (30 mmol, 3.6 g, 3.0 eq.) was added dropwise to the solution at 0 °C, and the mixture was stirred at room temperature for 20 min. After the reaction was completed, sat. NH_4_Cl was added to quench the reaction. The mixture was extracted with ethyl acetate, and the organic layer was washed with brine, dried over Na_2_SO_4_, and concentrated in vacuo. The residue was purified by trituration with *n*-hexane to obtain compound **11** as a brown solid (3.3 g, 73%); M.p.: 115–121 °C; ^1^H NMR (400 MHz, 298 K, DMSO-*d*_6_) δ 7.61 (d, *J* = 2.1 Hz, 2H), 7.38 (t, *J* = 2.1 Hz, 1H), 5.92–5.81 (m, 2H), 5.14 (dd, *J* = 8.5, 1.6 Hz, 2H), 5.10 (dd, *J* = 15.5, 1.6 Hz, 2H), 4.22 (d, *J* = 5.3 Hz, 4H), 1.39 (s, 18H); ^13^C NMR (100 MHz, 298 K, DMSO-*d*_6_) δ 167.1, 153.7, 143.7, 134.7, 131.8, 127.9, 124.1, 116.9, 80.7, 52.4, 28.3; IR (KBr): 3087, 2978, 2930, 2610, 1705, 1600, 1437, 1368, 1248, 1169, 877, 776 cm^−1^; HRMS (FAB) *m*/*z* [M+Na]^+^ calcd. for C_23_H_32_N_2_O_6_Na: 455.2158, found: 455.2158.

3,5-Bis(allylamino)benzoic acid (**8c**)

Compound **11** (6.0 mmol, 2.6 g) was dissolved in 6 M HCl, and the solution was stirred at room temperature for 20 h. After the reaction was completed, 4 M NaOH was added dropwise under cooling until the pH was 4. The mixture was extracted with ethyl acetate, and the organic layer was washed with brine, dried over Na_2_SO_4_, and concentrated in vacuo to obtain compound **8c** as an orange solid (1.3 g, 96%).; M.p.: 114–118 °C; ^1^H NMR (400 MHz, 298 K, DMSO-*d*_6_) δ 6.45 (d, *J* = 2.4 Hz, 2H), 6.01 (t, *J* = 2.1 Hz, 1H), 5.93–5.82 (m, 2H), 5.76 (t, *J* = 5.3 Hz, 2H), 5.25–5.17 (m, 2H), 5.12–5.05 (m, 2H), 3.63 (m, 4H); ^13^C NMR (100 MHz, 298 K, CDCl_3_) δ 169.0, 150.0, 136.8, 132.1, 115.7, 102.9, 100.3, 45.9; IR (KBr): 3396, 2978, 2845, 2625, 1686, 1604, 1520, 1460, 1333, 1309, 1215, 915 cm^−1^; HRMS (FAB) *m*/*z* [M+H]^+^ calcd. for C_13_H_17_N_2_O_2_: 233.1290, found: 233.1290.

General procedure (cyclization)

Triphenylphosphine and hexachloroethane (each 14.4 eq. to compound **9**) was added to a solution of the compound **8a**–**c** and benzene 1,3,5-tricarboxyric acid (**9**) at a 3:1 ratio in 1,1,2,2-tetrachloroethane, and the mixture was stirred at 120 °C with argon bubbling for 2 h. The reaction mixture was poured into water, extracted with dichloromethane, washed with brine, dried over Na_2_SO_4_, and concentrated in vacuo. The residue was purified by silica gel column chromatography and GPC.

*N*-Ethyl macrocycle (**1a**)

According to **GP**, compound **1a** was prepared by using compound **8a** (0.75 mmol, 0.16 g) and **9** (0.25 mmol, 50 mg) as monomers. The crude product was purified by silica gel column chromatography (chloroform, 5% MeOH) and GPC (chloroform). Compound **1a** was obtained as a white solid (30 mg, 16%). Optical resolution was performed by chiral high-performance liquid chromatography using CHIRALPAK IC (*n*-hexane–dichloromethane–acetonitrile–2-propanol = 12:4:1:1); M.p.: ≥300 °C; ^1^H NMR (400 MHz, 298 K, CDCl_3_) δ 6.99 (s, 3H), 6.82 (dd, *J* = 1.4, 1.0 Hz, 3H), 6.79 (dd, *J* = 1.8, 1.4 Hz, 3H), 6.68 (dd, *J* = 1.8, 2.1 Hz, 3H), 3.89–3.78 (m, 6H), 3.75–3.64 (m, 6H), 1.18 (t, *J* = 7.1 Hz, 9H), 1.15 (t, *J* = 7.1 Hz, 9H); ^13^C NMR (100 MHz, CDCl_3_) δ 169.0, 168.6, 144.2, 143.2, 140.7, 137.8, 131.1, 127.5, 127.0, 126.3, 45.3, 45.1, 12.9, 12.8; HRMS (FAB) *m*/*z* [M+H]^+^ calcd. for C_42_H_43_N_6_O_6_Na: 727.3244, found: 727.3244.

*N*-Benzyl macrocycle (**1b**)

According to **GP**, compound **1b** was prepared by using compound **8b** (1.5 mmol, 0.50 g) and **9** (0.5 mmol, 0.11 g) as monomers. The crude product was purified by silica gel column chromatography (ethyl acetate–dichloromethane = 1:2) and GPC (chloroform). Compound **1b** was obtained as a white solid (57 mg, 10%). Optical resolution was performed by chiral high-performance liquid chromatography using CHIRALPAK IA (*n*-hexane–dichloromethane = 1:1); M.p.: ≥300 °C; ^1^H NMR (400 MHz, 298 K, CDCl_3_) δ 7.34–7.28 (m, 18H), 7.10–7.04 (m, 6H), 7.02–6.97 (m, 6H), 6.86 (s, 3H), 6.39 (dd, *J* = 1.6, 1.6 Hz, 3H), 6.31 (dd, *J* = 1.8, 1.4 Hz, 3H), 5.92 (dd, *J* = 2.0, 1.8 Hz, 3H), 4.64 (m, 6H), 4.54 (s, 6H); ^13^C NMR (100 MHz, 298 K, CDCl_3_) δ 169.0, 168.3, 143.4, 142.9, 139.7, 137.5, 135.8, 135.6, 131.3, 129.4, 129.1, 128.95, 128.88, 128.4, 128.3, 127.5, 127.0, 126.0, 53.4, 53.3; IR (KBr): 3850, 3646, 2917, 1667, 1454, 704 cm^−1^; HRMS (APCI) *m*/*z* [M+H]^+^ calcd. for C_72_H_55_N_6_O_6_: 1099.4178; found: 1099.4180.

*N*-Allyl macrocycle (**1c**)

According to **GP**, compound **1c** was prepared by using compound **8c** (1.5 mmol, 0.35 g) and **11c** (0.5 mmol, 0.11 g) as monomers. The crude product was purified by silica gel column chromatography (chloroform, 3% MeOH) and GPC (chloroform). The compound **1c** was obtained as a white solid (53 mg, 13%); M.p.: ≥300 °C; ^1^H NMR (400 MHz, 298 K, CDCl_3_) δ 7.00 (s, 3H), 6.78 (dd, *J* = 1.5, 1.7 Hz, 3H), 6.76 (dd, *J* = 1.8, 1.4 Hz, 3H), 6.60 (dd, *J* = 2.0, 1.8 Hz, 3H), 5.92–5.77 (m, 6H), 5.24–5.16 (m, 6H), 5.13–5.04 (m, 6H), 4.53 (dd, *J* = 6.9, 7.3 Hz, 6H), 4.49 (dd, *J* = 7.3, 6.9 Hz, 6H), 4.02 (dd, *J* = 7.6, 7.8 Hz, 6H), 3.99 (dd, 6.6, 8.0 Hz, 6H); ^13^C NMR (100 MHz, 298 K, CDCl_3_) δ 169.0, 168.6, 143.9, 143.1, 140.2, 137.6, 131.7, 131.6, 131.3, 127.5, 127.0, 126.3, 120.6, 120.2, 52.9, 52.6; IR (KBr): 3066, 2984, 2935, 1651, 1587, 1439, 1401, 1323, 1207, 928, 709 cm^−1^; HRMS (FAB) *m*/*z* [M+H]^+^ calcd. for C_48_H_43_N_6_O_6_: 799.3244; found: 799.3244.

## 4. Conclusions

A new synthetic approach to access a structural isomer of spherical amides was developed. This method, which includes *N*-alkylation and one-step cyclization, demonstrated advantages over the previous method in terms of reaction time and experimental simplicity. Furthermore, the developed approach enabled the introduction of novel substituents, such as benzyl and allyl groups, into spherical amides. ^1^H NMR spectra and X-ray crystallographic analysis of **1b** (isomer **1**, *N*-benzyl derivative) confirmed that the core structure possessed a spherical shape with *C*_3_ symmetry. In the crystal structure, two independent molecules with the same chirality were arranged alternately to form 1D columnar structures. The absolute structure of this isomer was determined via Flack parameter analysis. On the basis of this result, the absolute structure of other spherical amides with different substituents can potentially be determined by comparing their ECD spectra because the Cotton effects originating from the core structure did not depend on substituents. The high-symmetry *C*_3_ structure of isomer **1** resulted in larger ECD values and higher crystallinity compared to those of isomer **2**, suggesting that this core structure is suitable as a scaffold for chiroptical emitters or crystalline materials. Future research will focus on functionalizing spherical amides to explore their molecular recognition ability, chiroptical properties, and bioactivity.

## Figures and Tables

**Table 1:** Torsion angles of molecule A and B in a crystal of (+)-1b.

	∠C(=O)-N-C(H_2_)-C(sp^2^) [°]	
	Molecule A	Molecule B
*θ*_1_	94.4(5)	89.8(5)
*θ*_2_	−100.4(5)	−128.7(5)
*θ*_3_	104.1(5)	103.7(5)
*θ*_4_	−96.1(5)	−112.6(6)
*θ*_5_	−102.2(4)	−73.7(6)
*θ*_6_	124.3(4)	97.7(5)

## Data Availability

Data will be made available on request.

## Supplementary Materials

The following supporting information can be downloaded at: https://www.mdpi.com/article/10.3390/molecules30051074/s1, S1—^1^H and ^13^C NMR spectra, S2—2D NMR spectra of *N*-benzyl macrocycle (1b), S3—Chiral separation, S4—X-ray crystallographic analysis, S5—References; Figure S1—Charts of chiral HPLC and analytical conditions of chiral separation, Figure S2—ORTEP diagram of molecule A and B in a crystal of enantiopure (+)-**1b**, Figure S3—Superimposed structures of (+)-**1a**; Table S1—Torsion angles in the crystals. References [47,48] are cited in Supplementary Materials.
