# Supplementary material for: Spherical Amides with C3 Symmetry: Improved Synthetic Approach and Structural/Optical Analysis
Source: Molecules. 2025 Feb 26;30(5):1074. doi: 10.3390/molecules30051074 (PMC11901929; doi:10.3390/molecules30051074)
Supplement: Supplementary file 1 [file molecules-30-01074-s001.zip › molecules-3480880-supplementary.pdf]

Supplementary Materials

Spherical Amides with C_3 Symmetry: Improved Synthetic Approach and Structural/Optical Analysis

Daki Koike [1], Hyuma Masu [2], Haruka Uno [1], Shoko Kikkawa [1], Hidemasa Hikawa [1] and Isao Azumaya [1,*]

[1] Faculty of Pharmaceutical Sciences, Toho University, 2-2-1 Miyama, Funabashi, Chiba 274-8510, Japan
[2] Center for Analytical Instrumentation, Chiba University, 1-33 Yayoi-cho, Inage-ku, Chiba 263-8522, Japan
* Correspondence: isao.azumaya@phar.toho-u.ac.jp; Tel.: +81-47-472-1589

S1. ^{1}H and ^{13}C NMR spectra

Ethyl 3,5-bis(benzylamino)benzoate (7b)

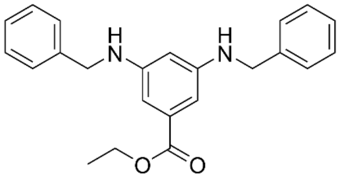

^{1}H NMR (400 MHz, $CDCl_3$)

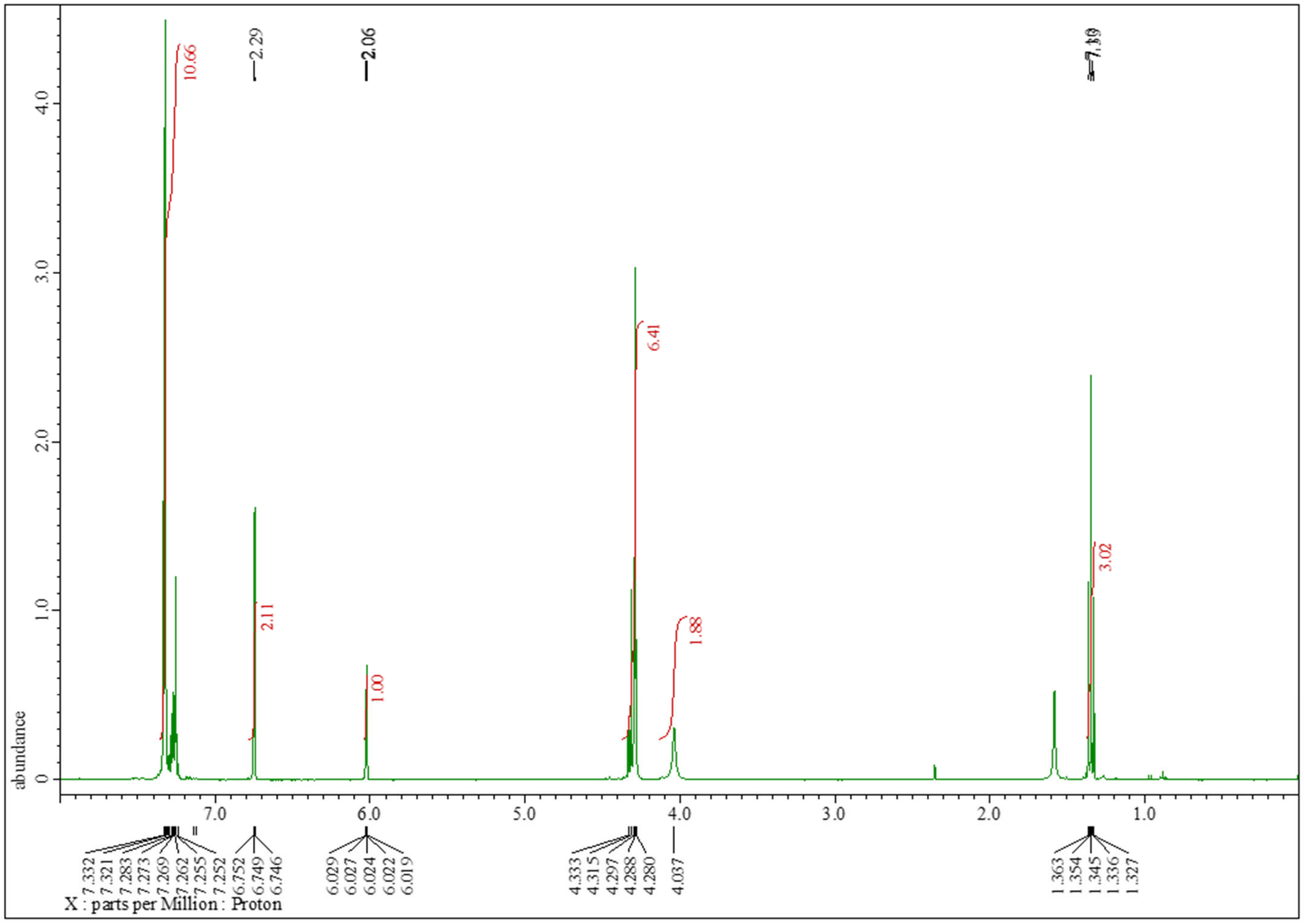

^{13}C NMR (100 MHz, $CDCl_3$)

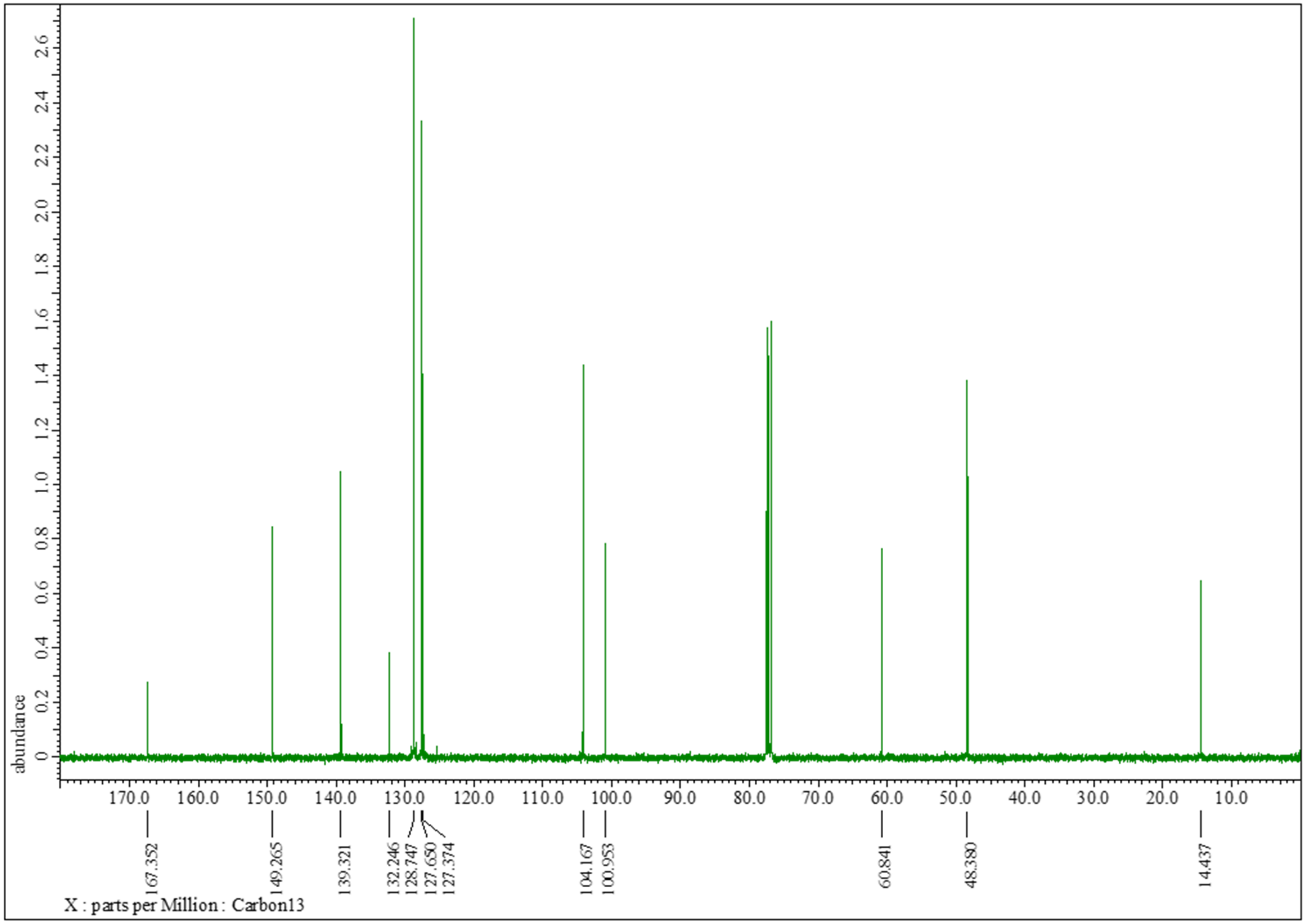

3,5-Bis(benzylamino)benzoic acid (8b)

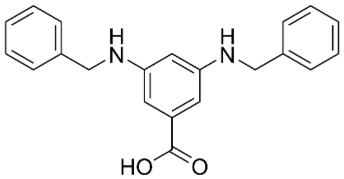

^{1}H NMR (400 MHz, DMSO-d_6)

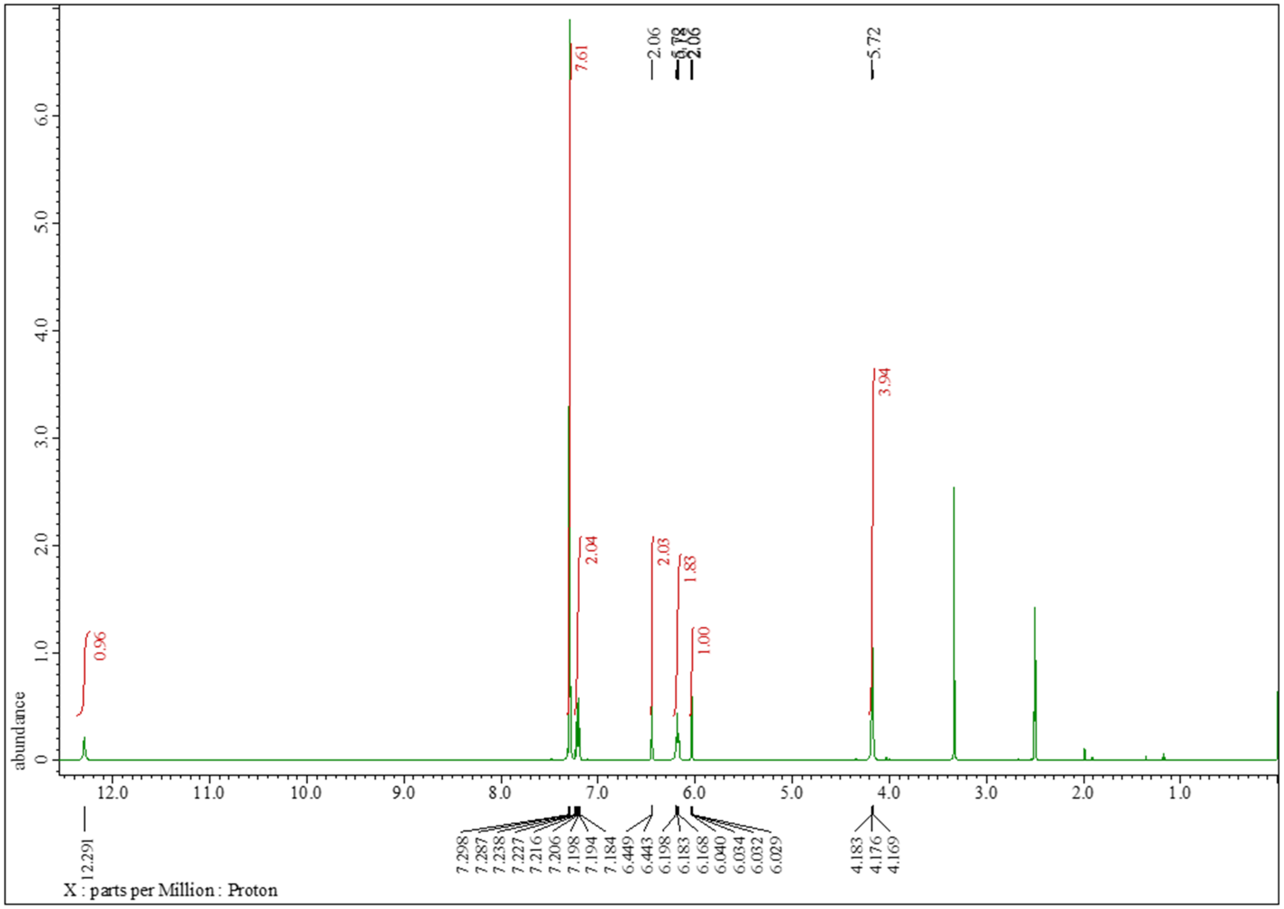

^{13}C NMR (100 MHz, DMSO-d_6)

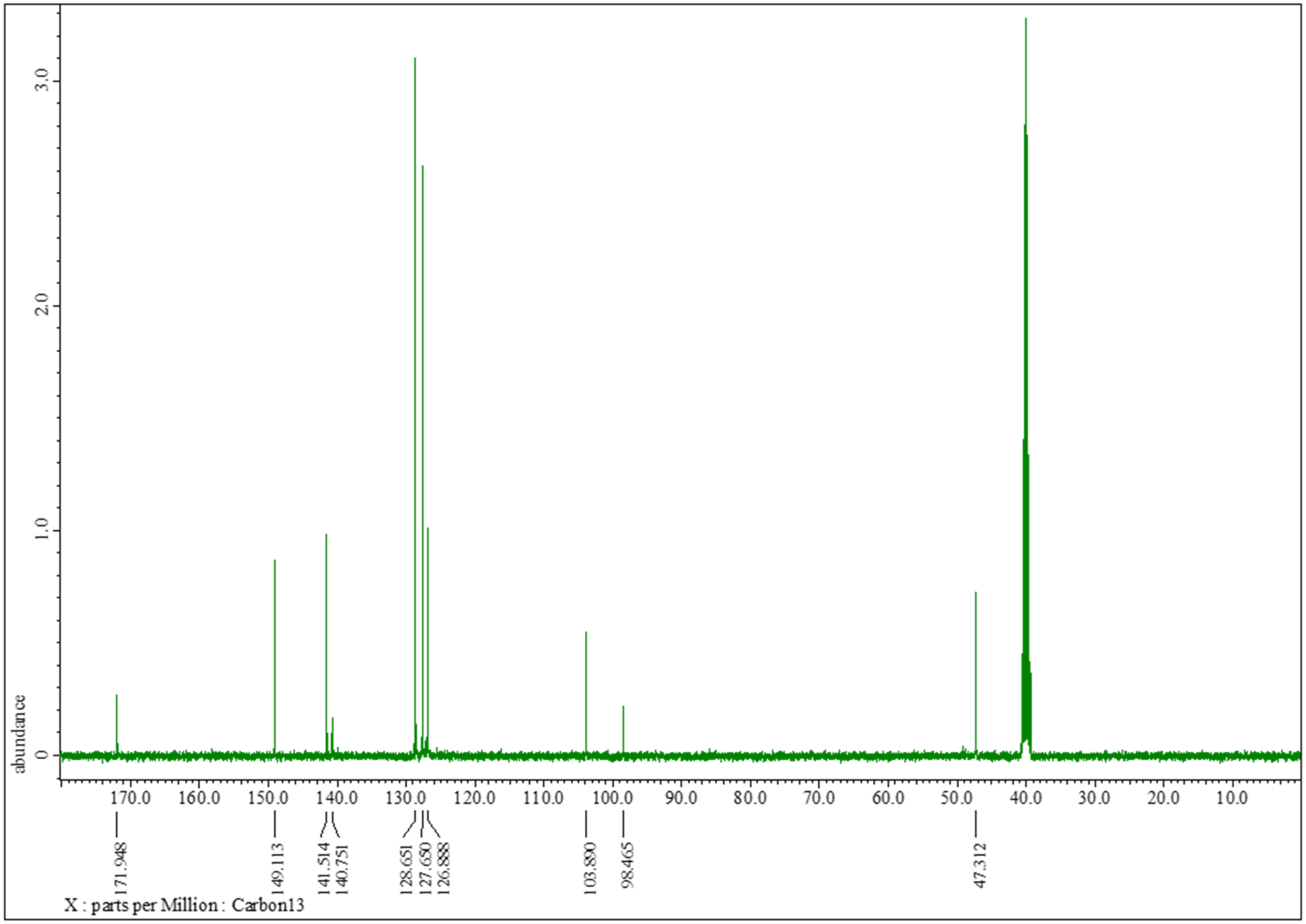

3,5-Bis[(*tert*-butoxycarbonyl)amino]benzoic acid (10)

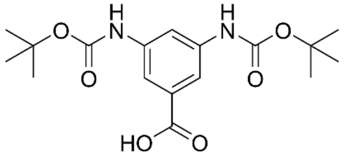

^{1}H NMR (400 MHz, DMSO-d_6)

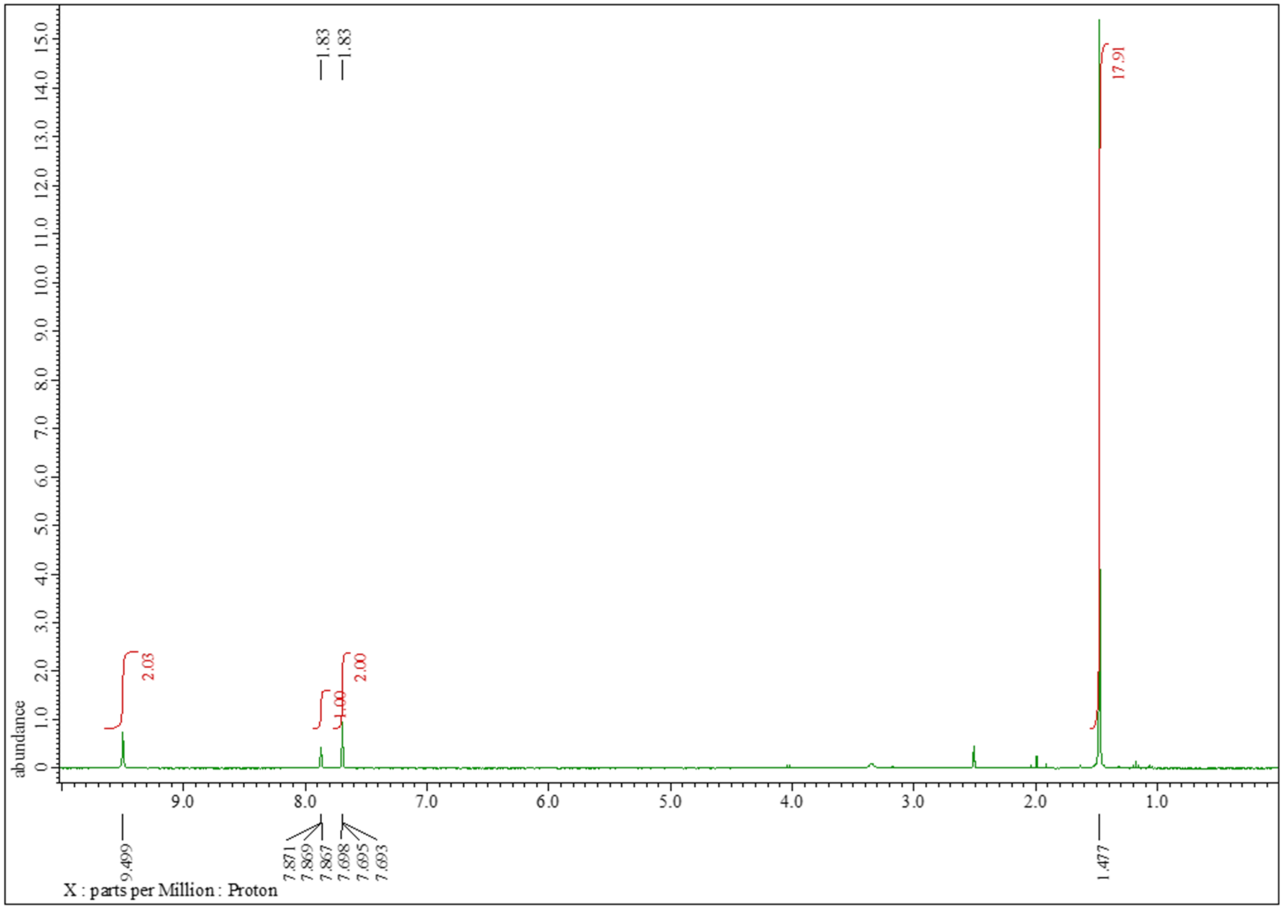

^{13}C NMR (100 MHz, DMSO-d_6)

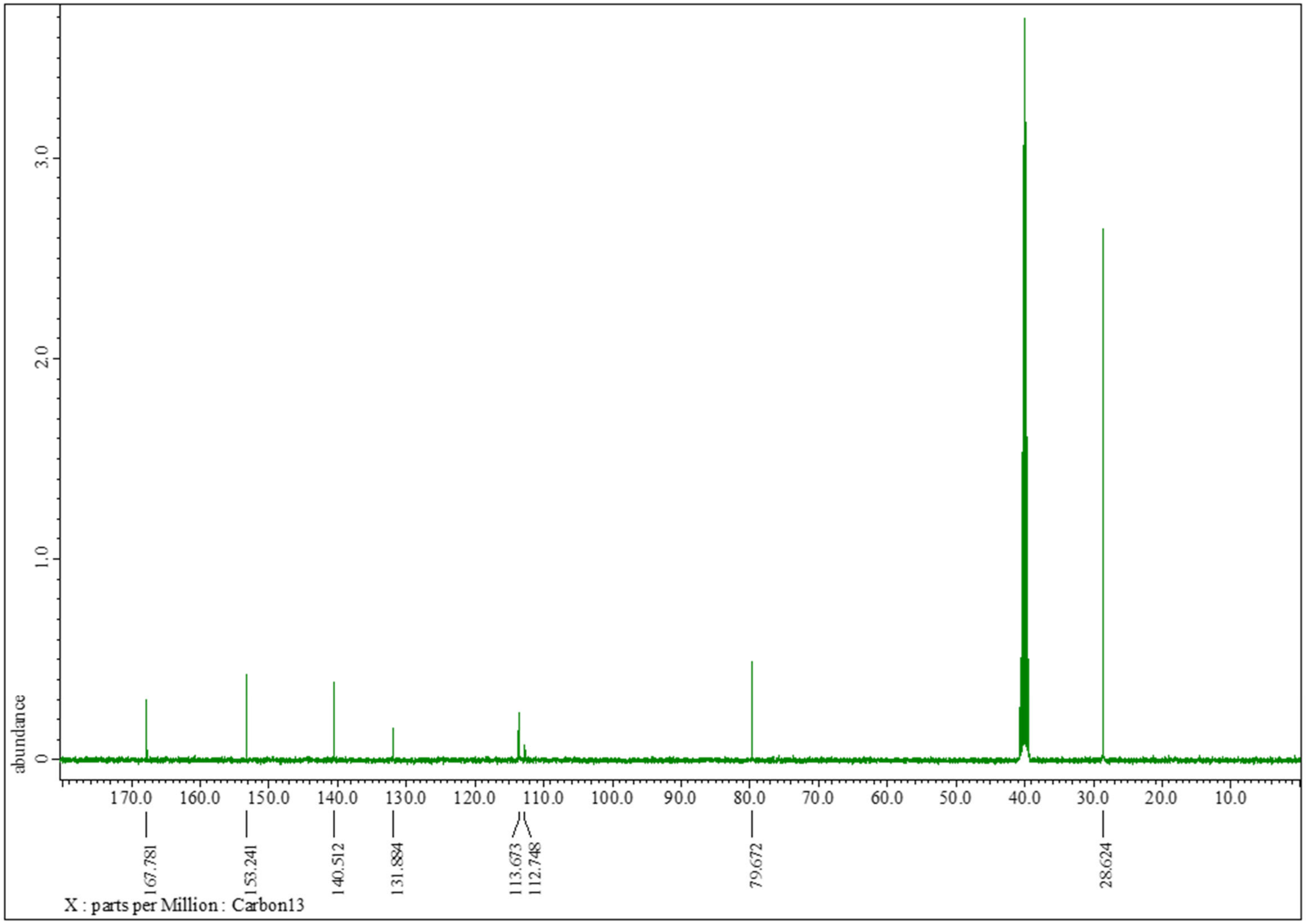

3,5-Bis[allyl(*tert*-butoxycarbonyl)amino]benzoic acid (11)

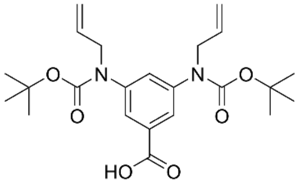

^{1}H NMR (400 MHz, DMSO-d_6)

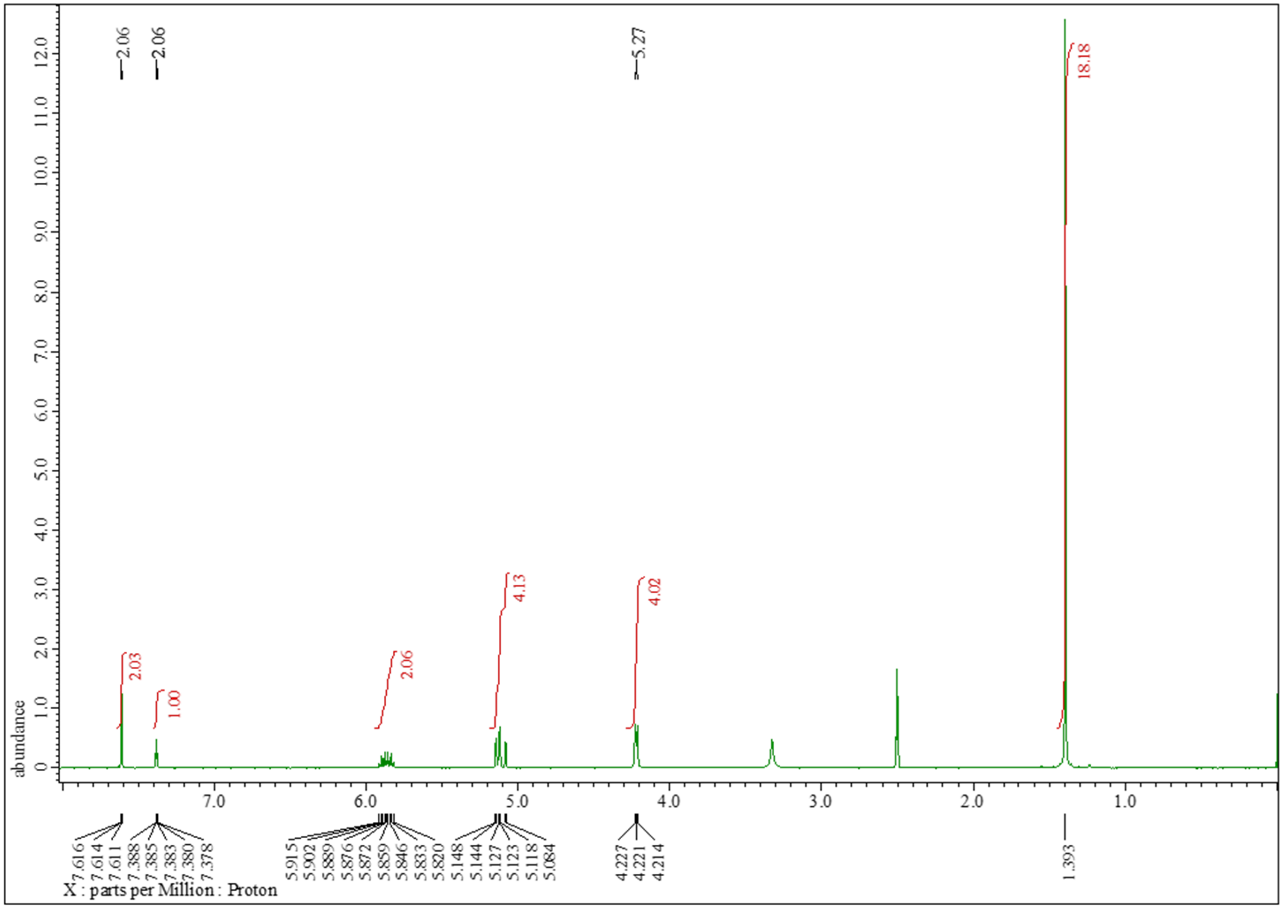

^{13}C NMR (100 MHz, DMSO-d_6)

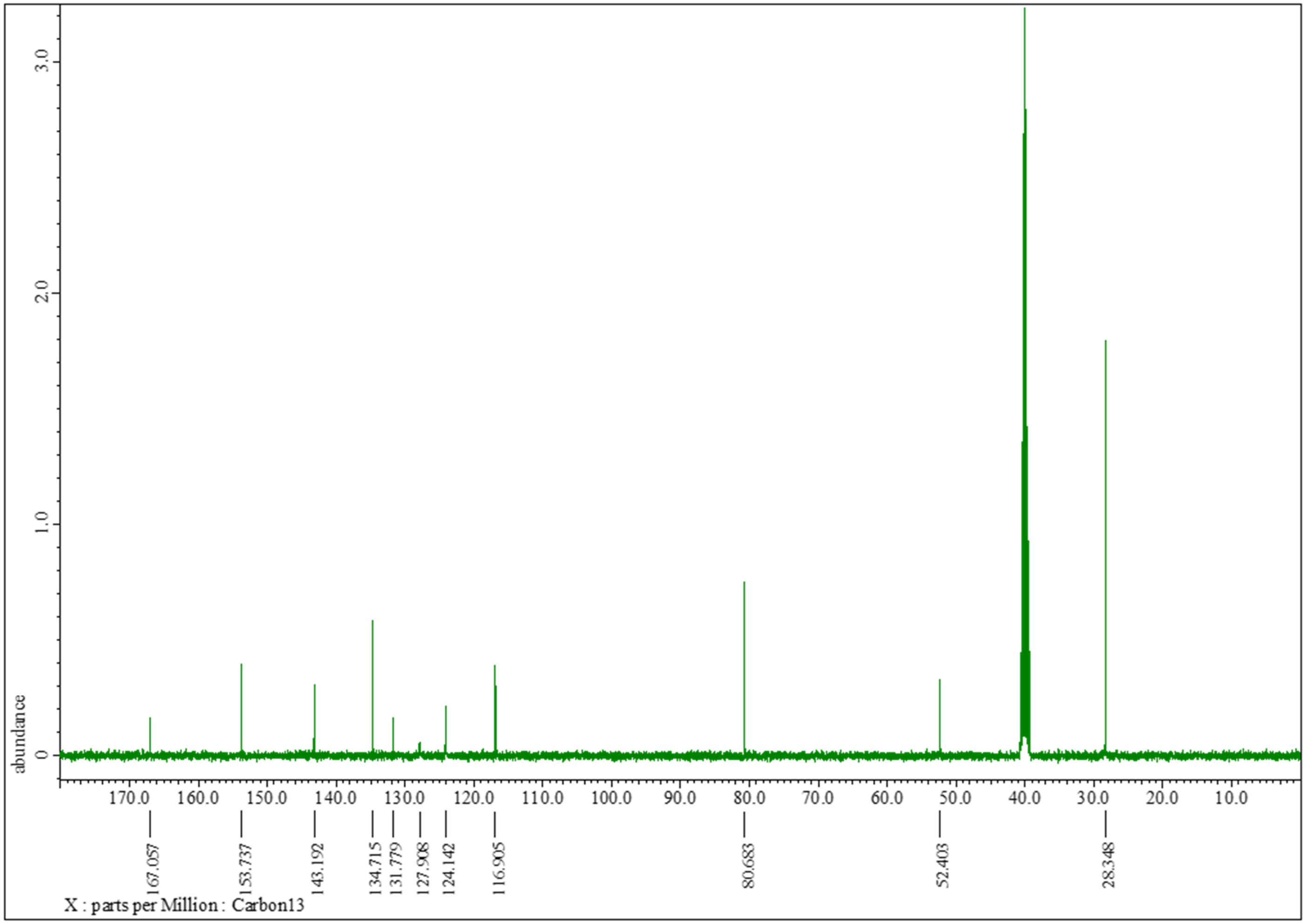

3,5-Bis(allylamino)benzoic acid (8c)

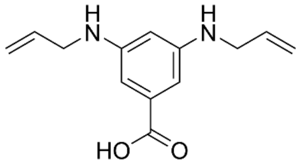

^{1}H NMR (400 MHz, DMSO-d_6)

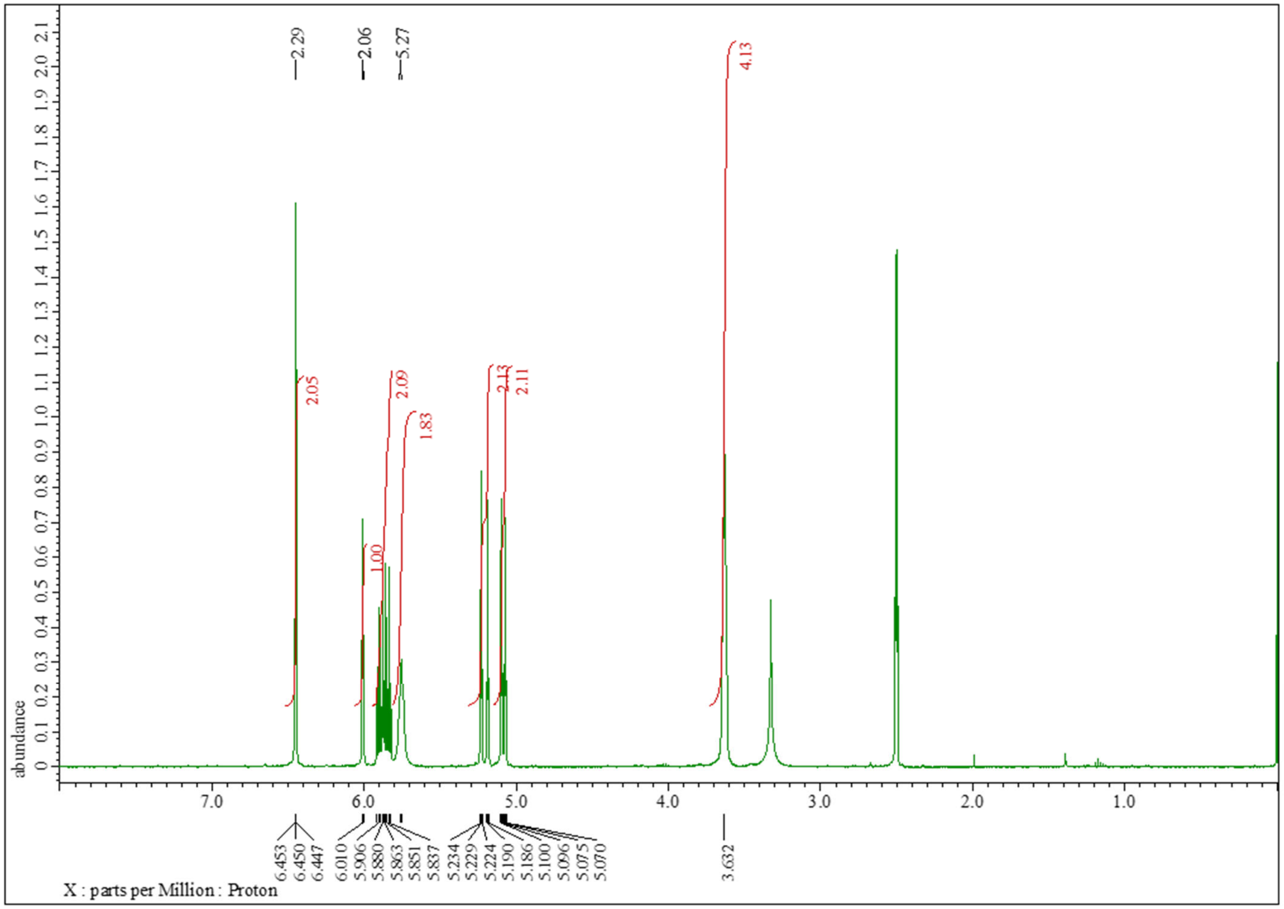

^{13}C NMR (100 MHz, DMSO-d_6)

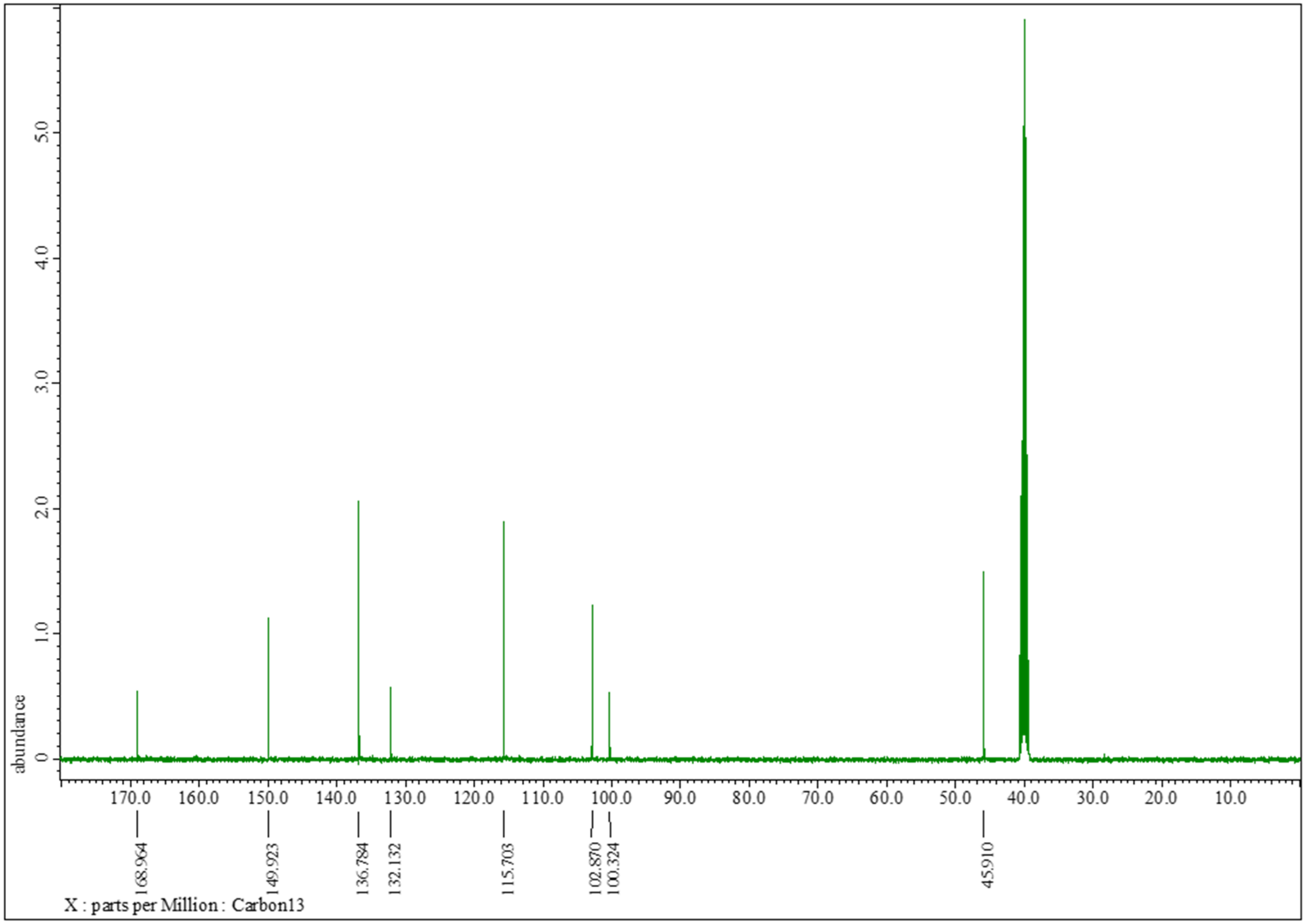

N-Ethyl Macrocycle (1a)

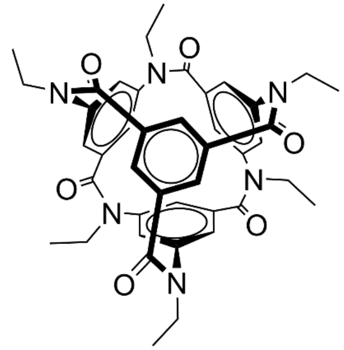

^{1}H NMR (400 MHz, $CDCl_3$)

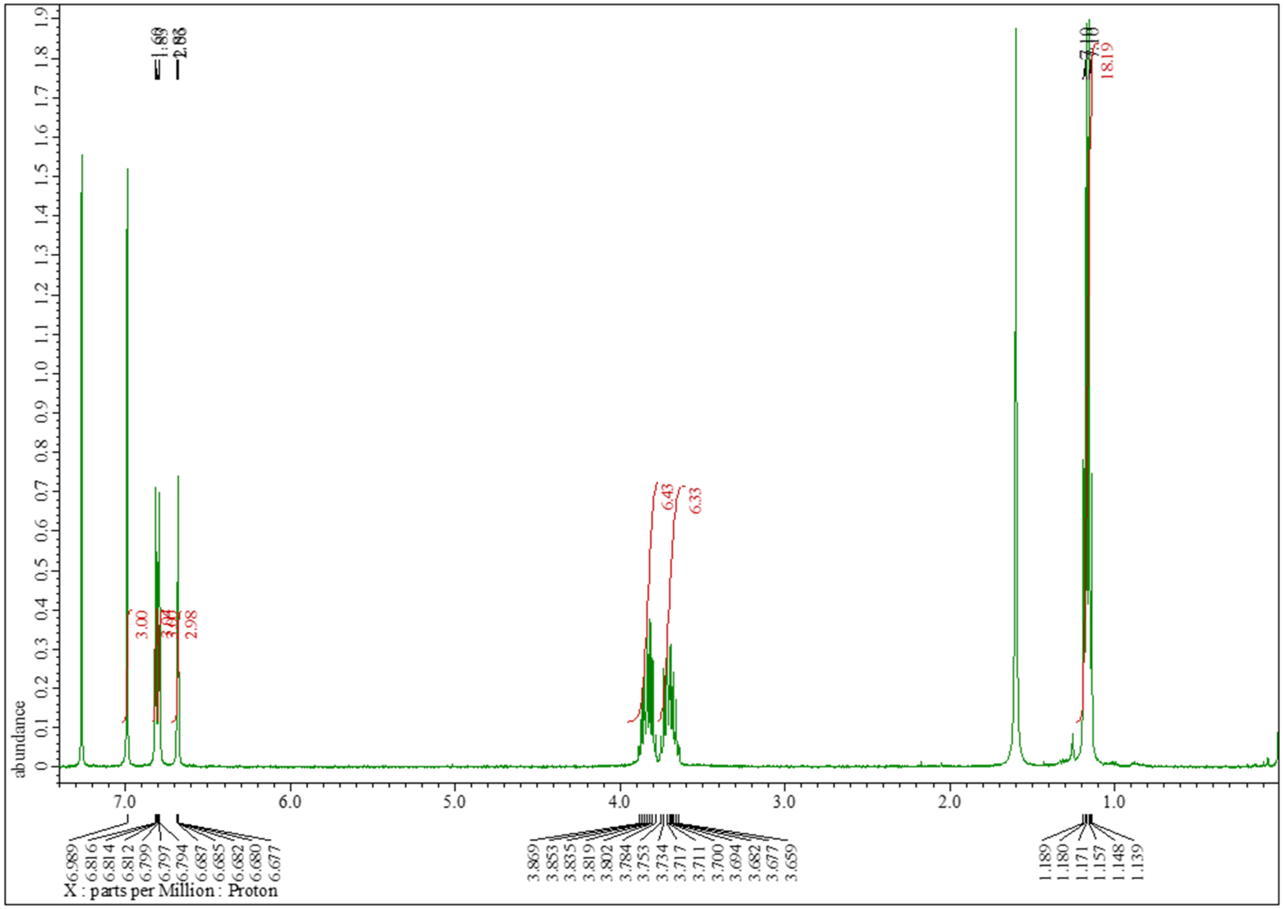

^{13}C NMR (100 MHz, $CDCl_3$)

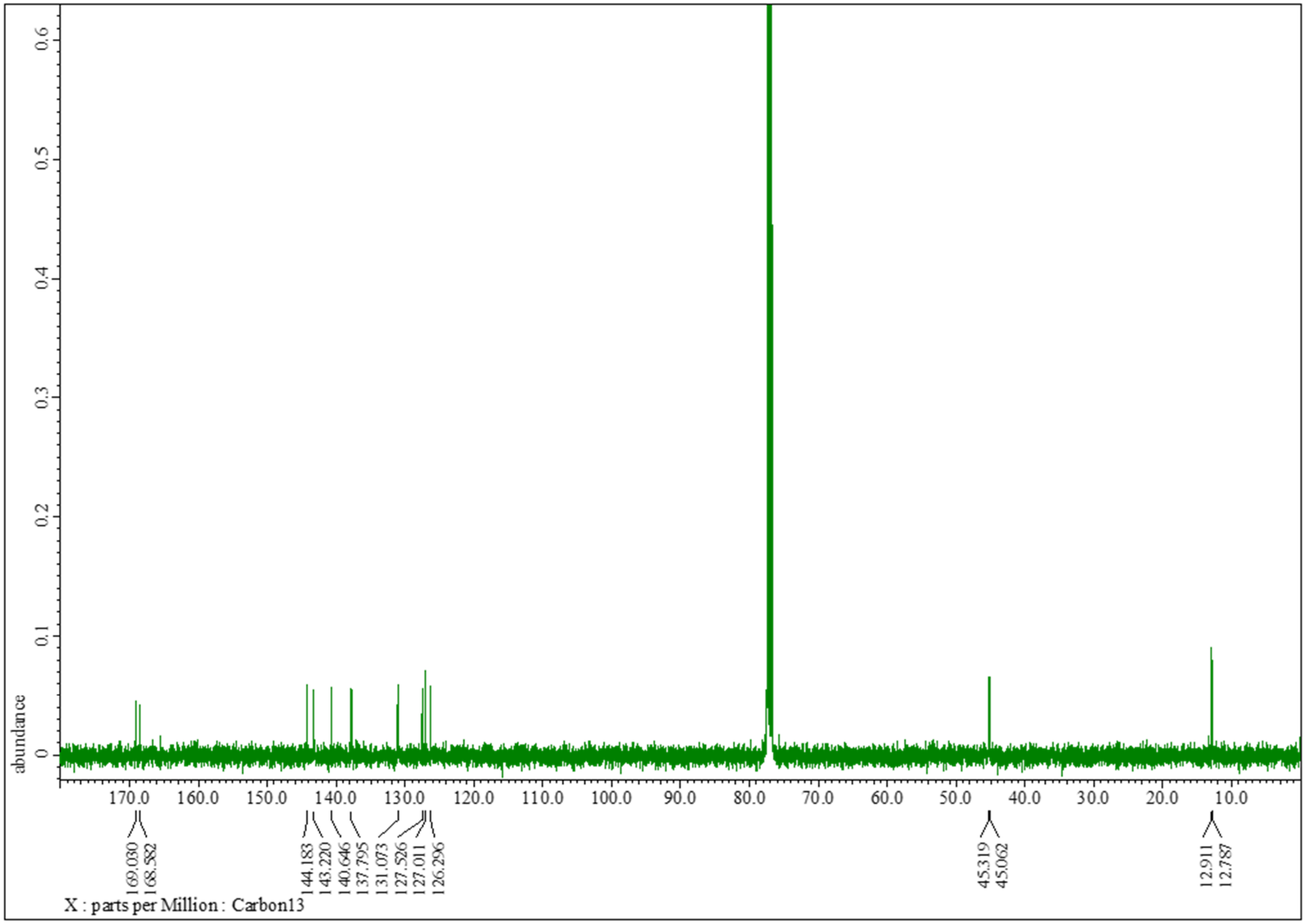

N-Benzyl Macrocycle (1b)

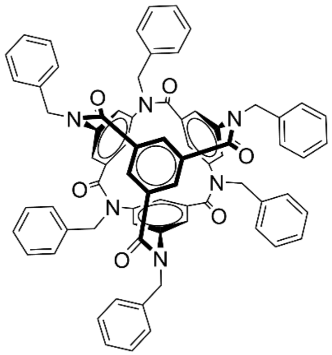

^{1}H NMR (400 MHz, $CDCl_3$)

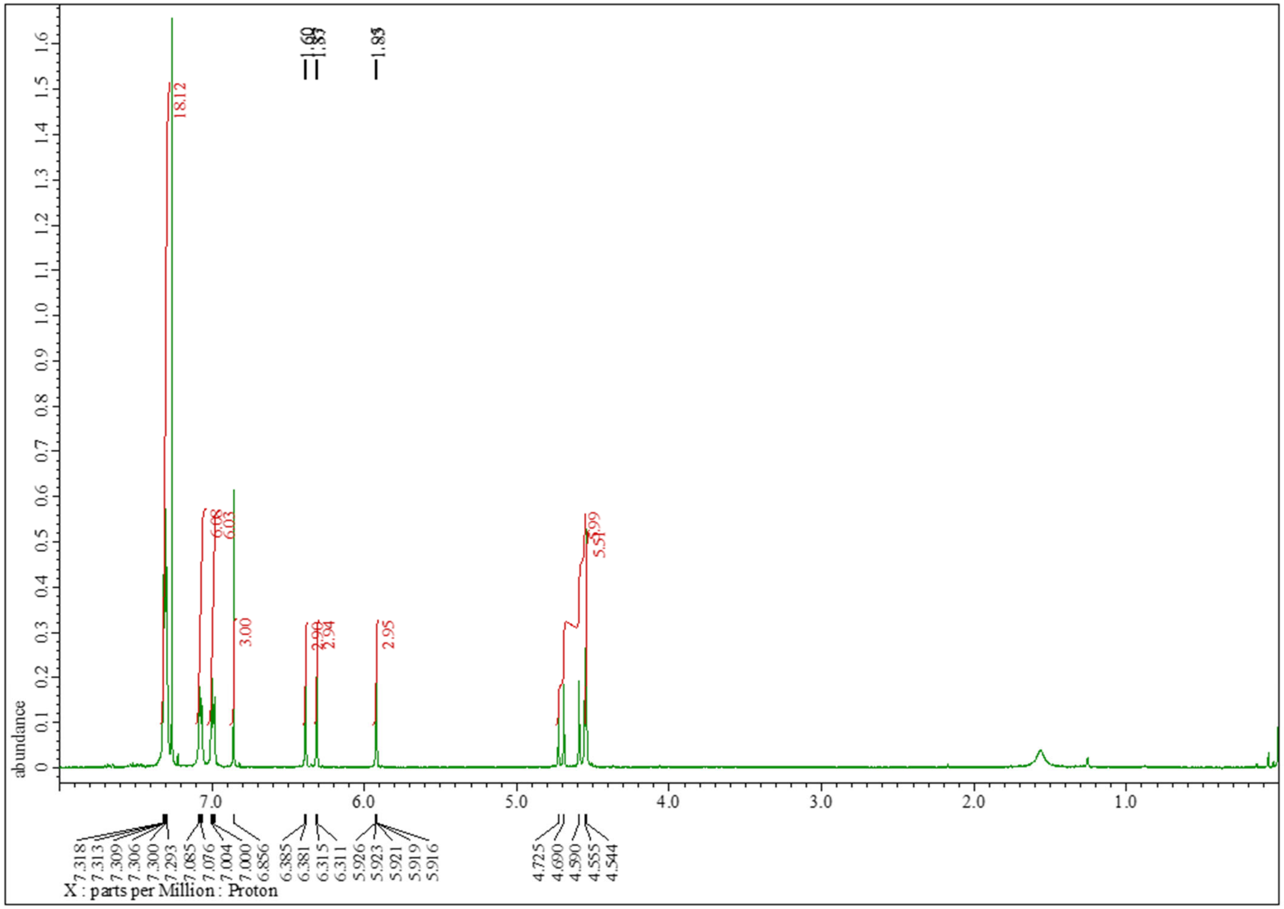

^{13}C NMR (100 MHz, $CDCl_3$)

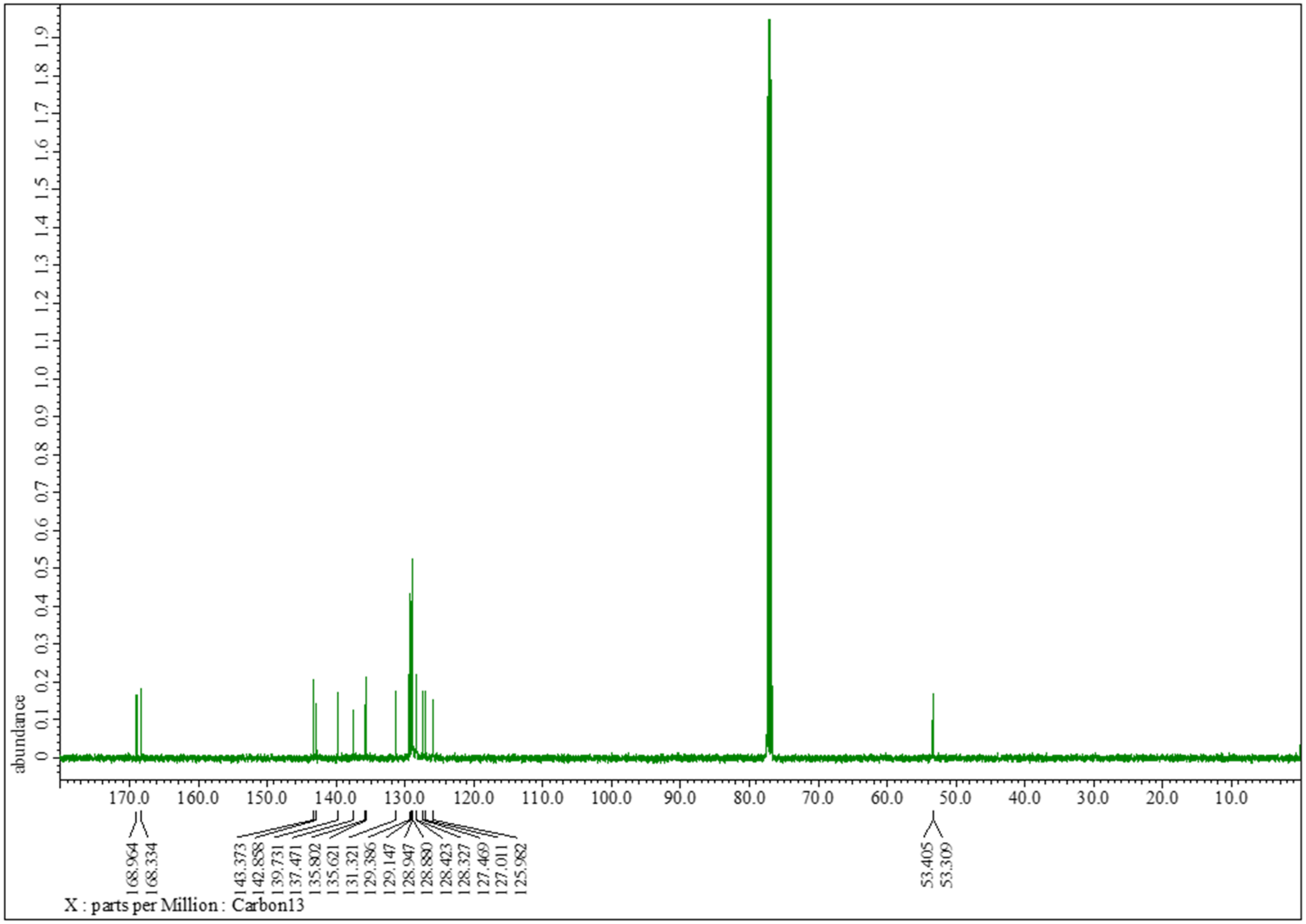

N-Allyl Macrocycle (1c)

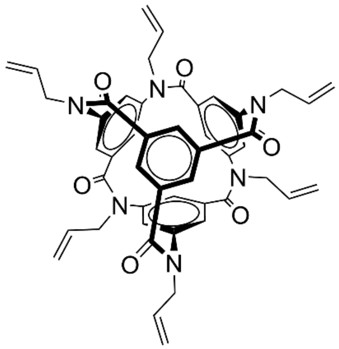

^{1}H NMR (400 MHz, $CDCl_3$)

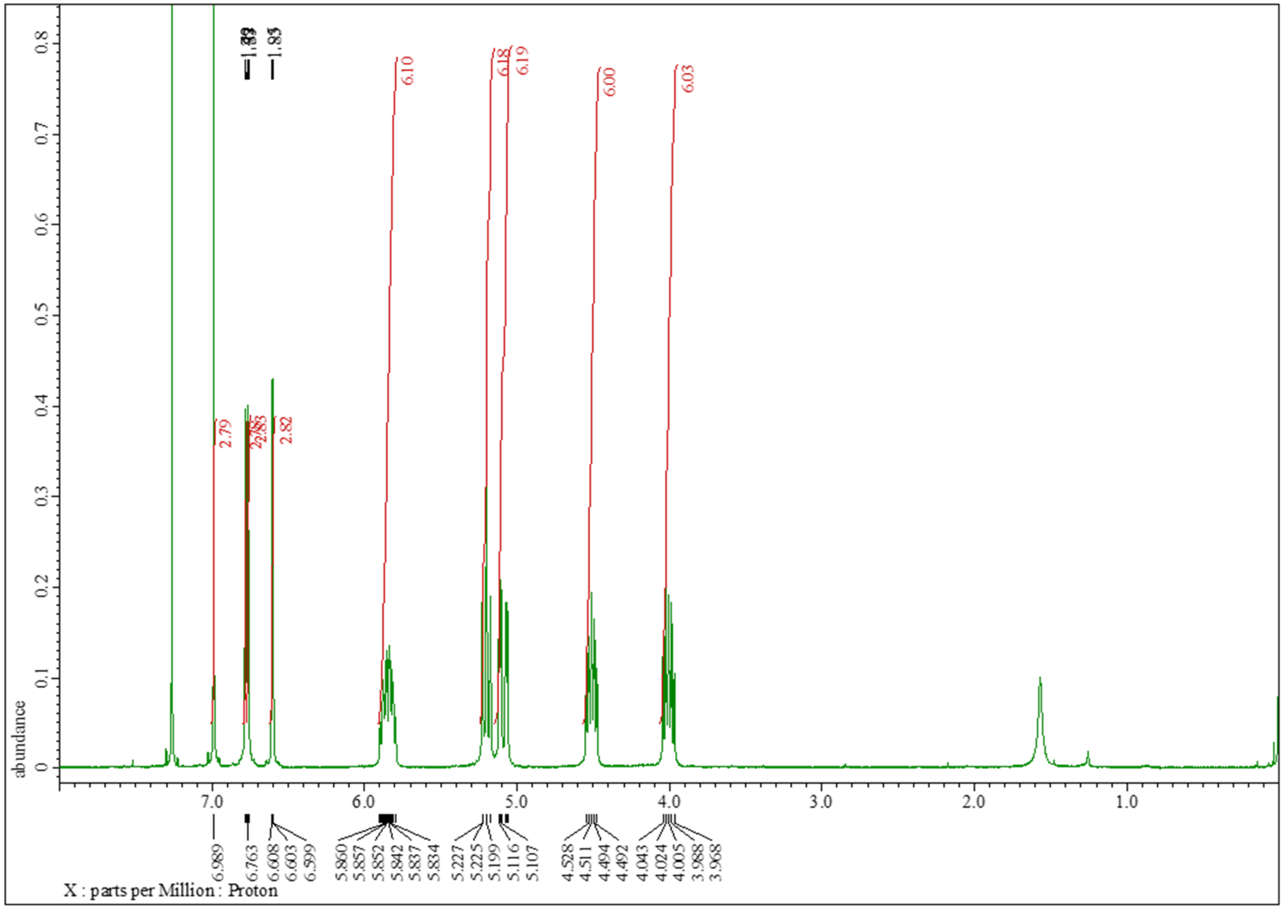

^{13}C NMR (100 MHz, $CDCl_3$)

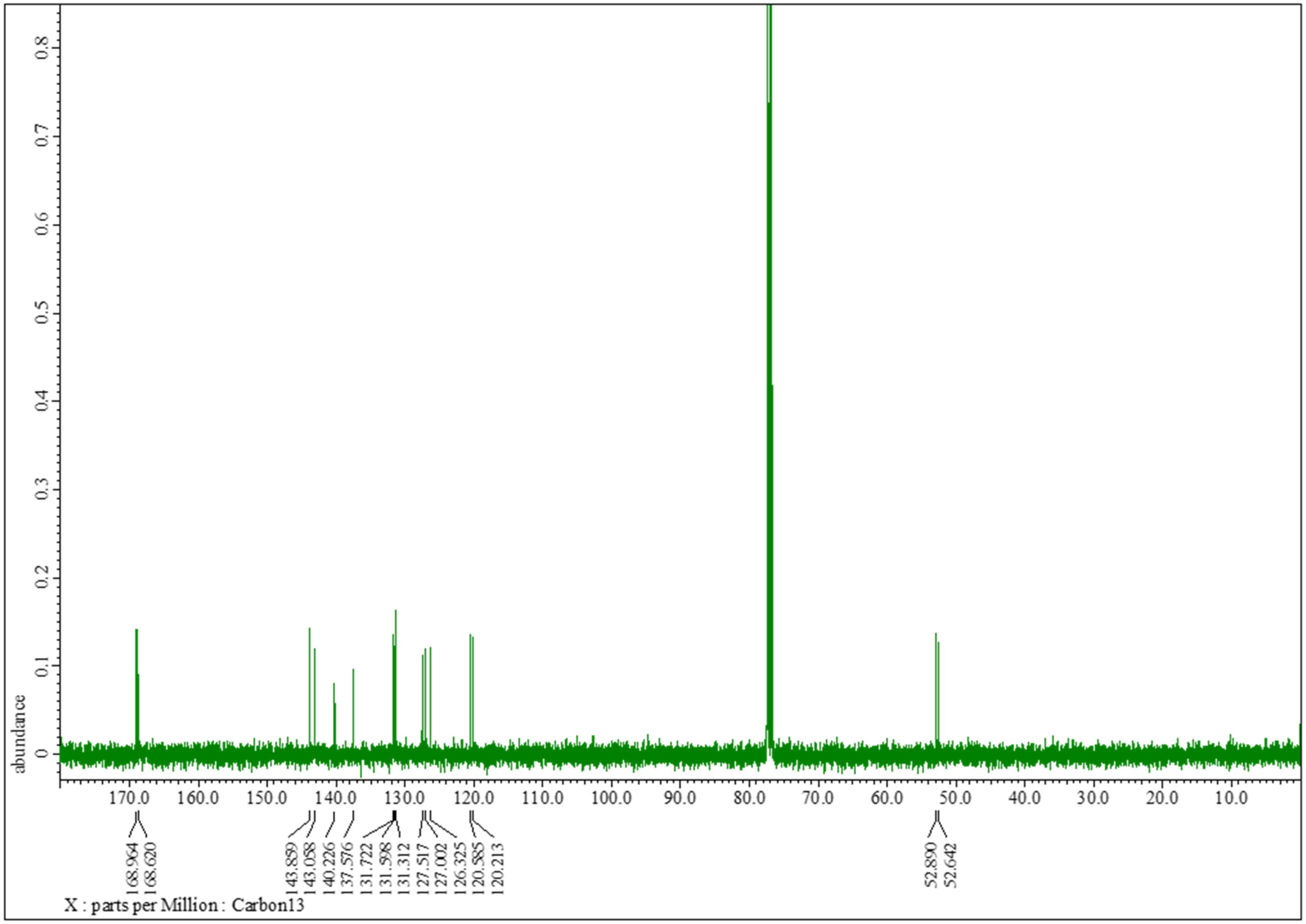

S2. 2D NMR spectra of *N*-benzyl macrocycle (1b)

^{1}H-^{1}H COSY NMR ($CDCl_3$)

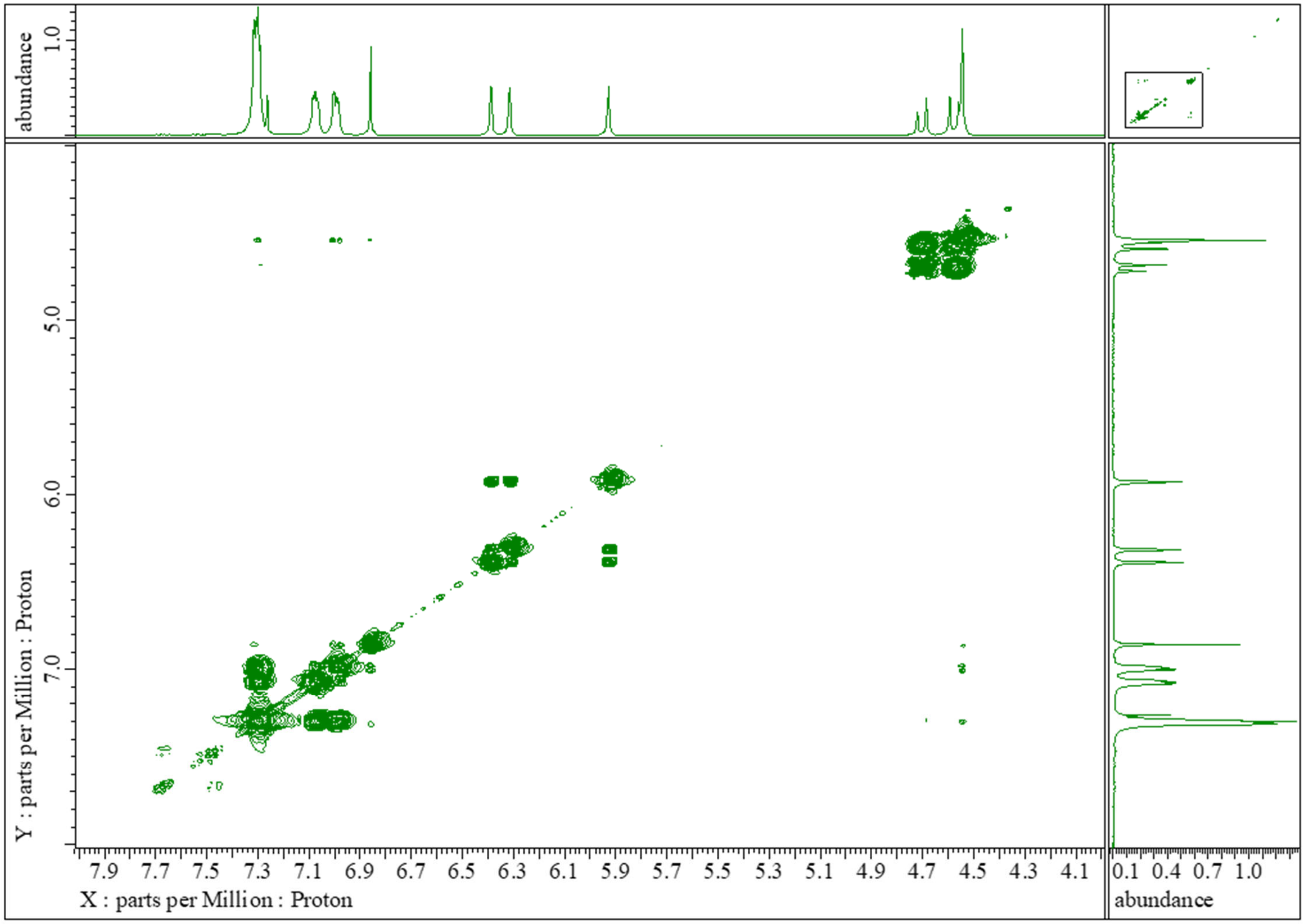

^{1}H-^{13}C HMBC NMR ($CDCl_3$)

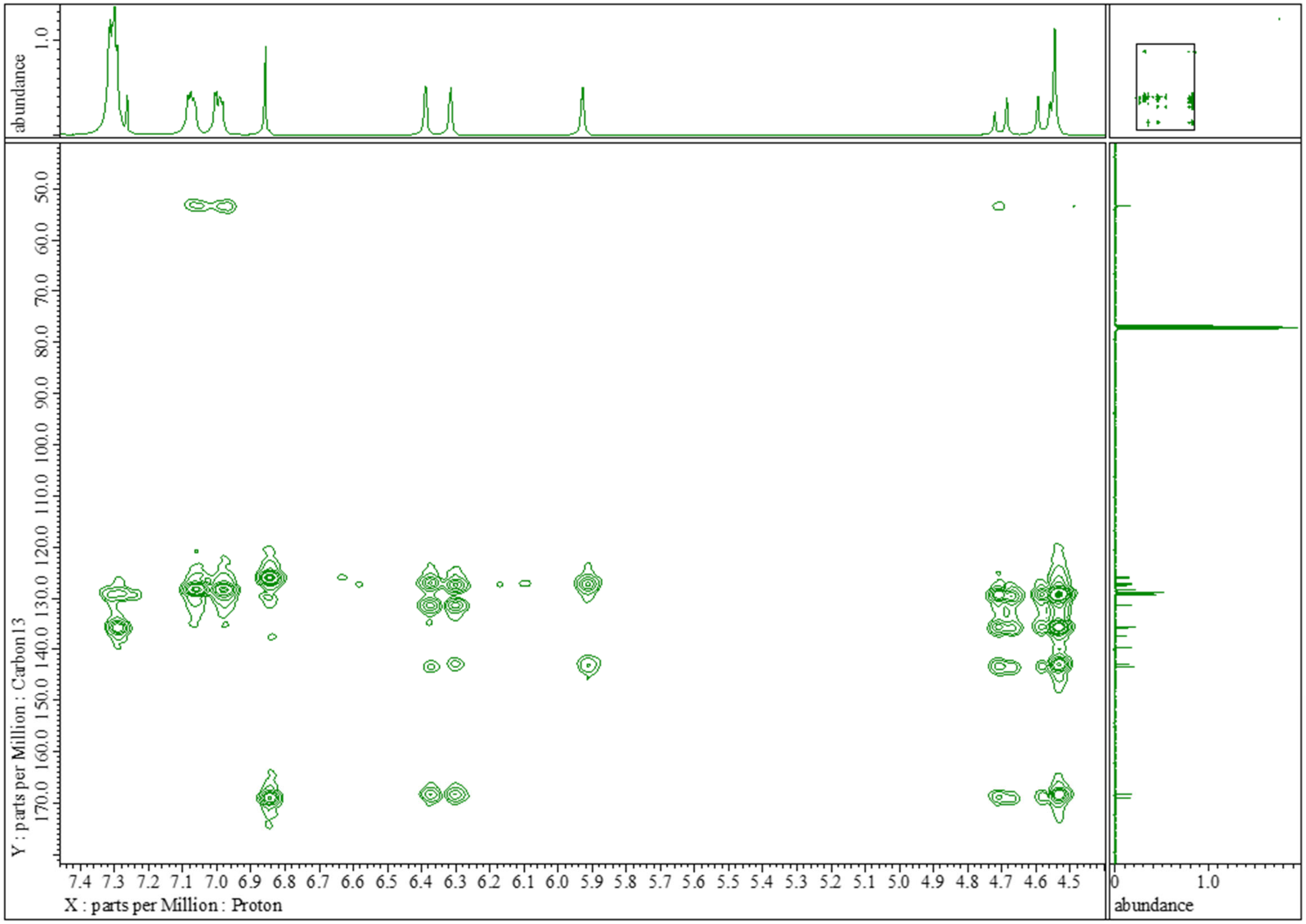

^{1}H-^{13}C HSQC NMR ($CDCl_3$)

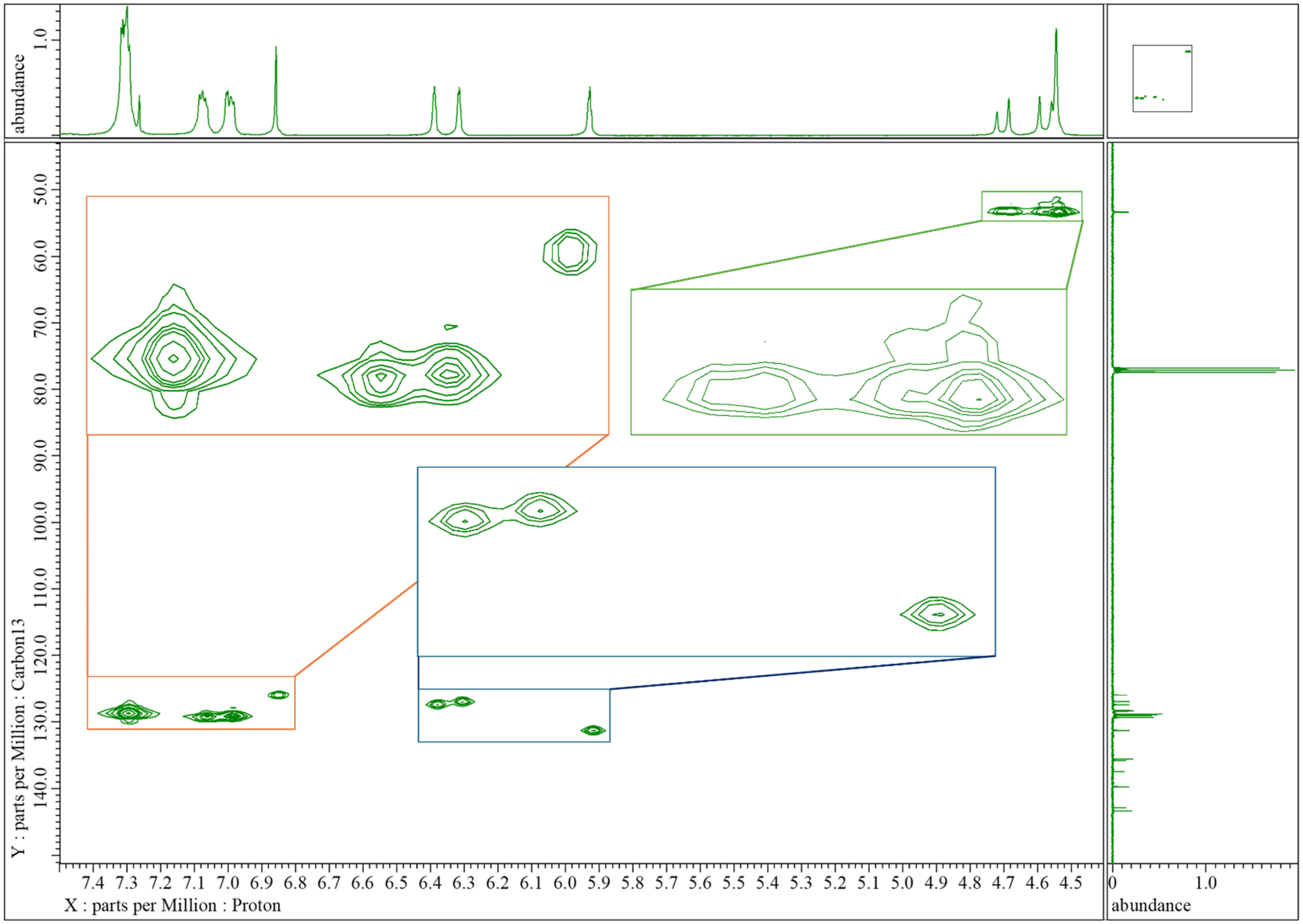

^{1}H-^{1}H NOESY NMR ($CDCl_3$)

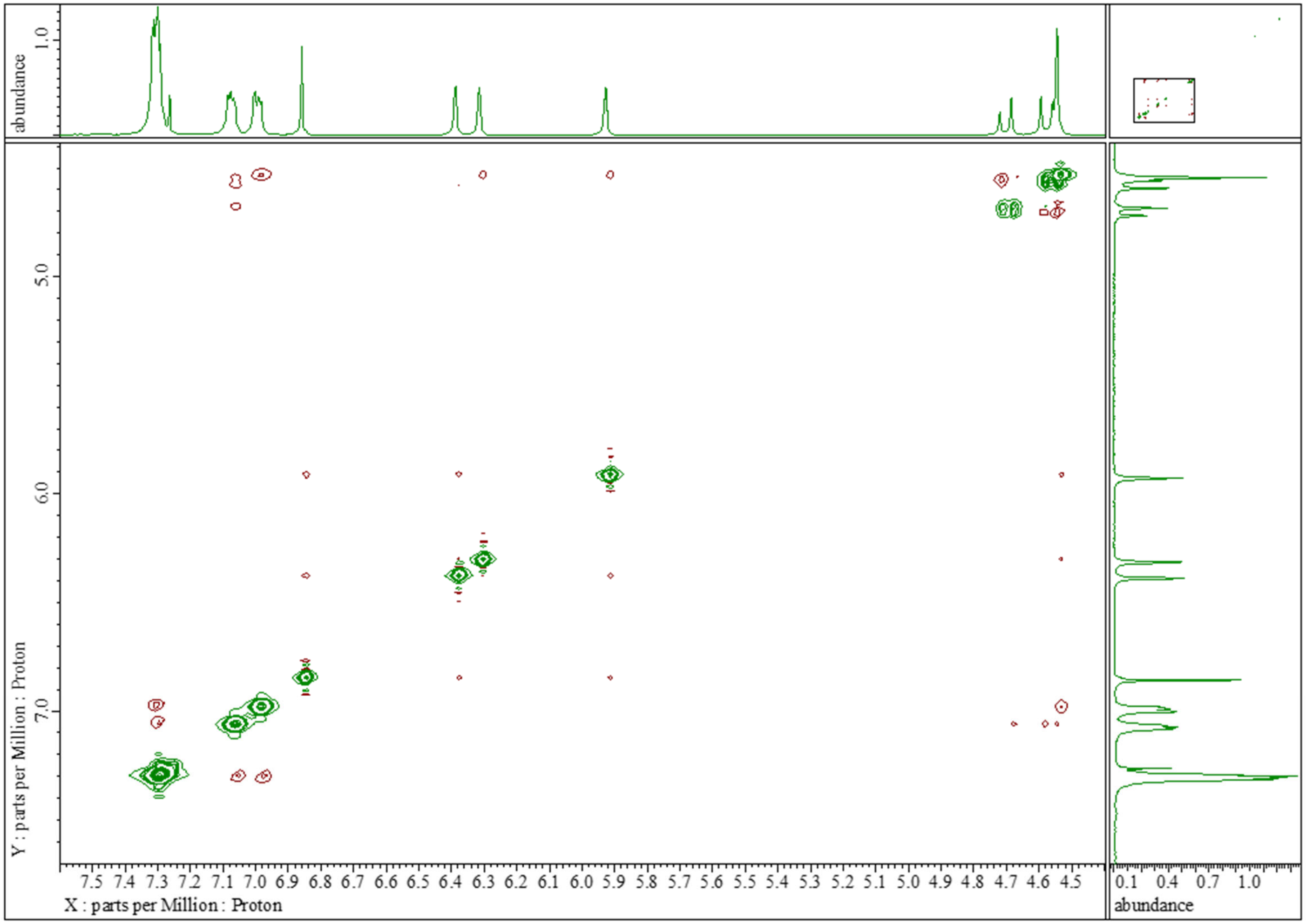

S3. Chiral separation

The chiral separations of **1a**, **1b** and **1c** were carried out by chiral HPLC with DAICEL CHIRALPAK column. Other details in analytical condition are described in Figure S5. For preparation, semi-preparative column (10 mmφ × 250 mm) of the same stationary phase was used and the flowrate in each preparation was increased to 4.7 mL/min. The enantiomeric purity of each compound was 100% e.e.

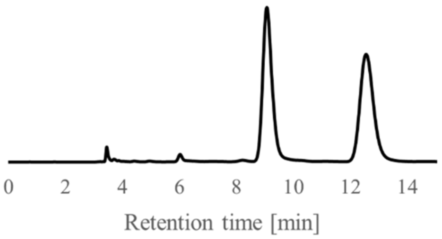

1a (*N*-ethyl)

Column: CHIRALPAK IC (2.1 mmφ × 250 mm)
Solvent: *n*-Hexane/Dichloromethane/Acetonitrile/2-Propanol
= 12 : 4 : 2 : 1
Flowrate: 1.0 mL/min

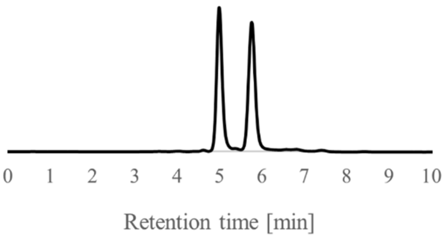

1b (*N*-benzyl)

Column: CHIRALPAK IA (2.1 mmφ × 250 mm)
Solvent: *n*-Hexane/Dichloromethane
= 1 : 1 (2-Propanol: 1%)
Flowrate: 1.0 mL/min

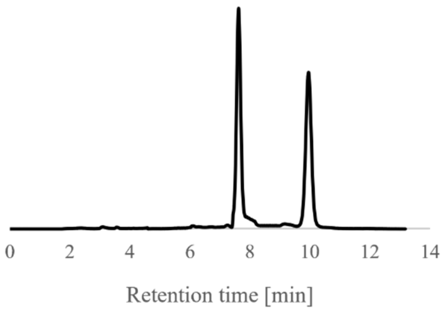

1c (*N*-allyl)

Column: CHIRALPAK IC (2.1 mmφ × 250 mm)
Solvent: Acetonitrile/*n*-Hexane/Dichloromethane
= 2 : 1 : 1
Flowrate: 1.0 mL/min

Figure S1. Charts of chiral HPLC and analytical conditions of chiral separation.

S4. X-ray crystallographic analysis

X-ray data were collected on and on a Rigaku XtaLAB P200 diffractometer with multi-layer mirror monochromated Mo$K\alpha$ (λ = 0.71075 Å) and a hybrid photon counting detector (PILATUS 200K). The crystal structure was solved by direct methods (SHELXT Version 2014/5)[S1] and refined by full-matrix

least-squares SHELXL-2014/7.[S2]

Crystallographic data for (+)-**1b**: $C_{74}H_{58}Cl_4N_6O_{6.5}$, M_r = 1277.12, 0.293 × 0.200 × 0.040 mm, monoclinic, $P2_1$ (no. 4), a = 17.9706(6), b = 18.2862(5), c = 19.7126(6) Å, b = 101.913(3) º, V = 6338.3(3) Å^3, Z = 4, $D_{calcd.}$ = 1.338 gcm^{-3}, θ_{max} = 27.498, T = 93 K, 106925 reflections measured, 28625 unique (R_{int} = 0.0615), μ = 0.248 mm^{-1}, T_{max} = 0.952, T_{min} = 0.780. The final R_1 and wR_2 were 0.0617 and 0.1637 (all data). The residual electron densities (peak and hole) were 1.44 and -0.61 eÅ^{-3}. All non-H-atoms were refined anisotropically, and H-atoms were fixed in geometrically estimated positions and refined using the riding model. The crystal consists of two independent molecular conformations (molecule A, B) per asymmetric unit with solvent molecules (CH_2Cl_2 and H_2O). Molecule A is the one containing benzene rings numbered C1-C6, and Molecule B is the one containing benzene rings numbered C101-C106. CCDC 2419806 contain the supplementary crystallographic data for this paper. These data can be obtained free of charge from The Cambridge Crystallographic Data Centre via https://www.ccdc.cam.ac.uk/structures/.

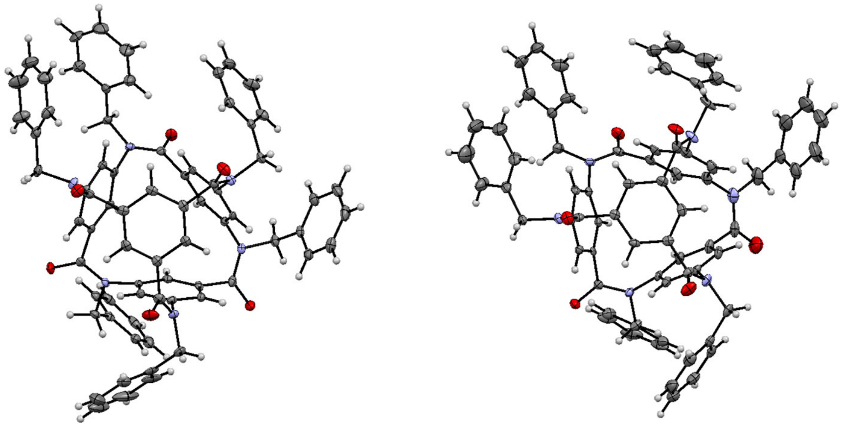

(A) (B)

Figure S2. ORTEP diagram of molecule A and B in a crystal of enantiopure (+)-**1b**. The ellipsoids of non-hydrogen atoms are drawn at the 50% probability level.

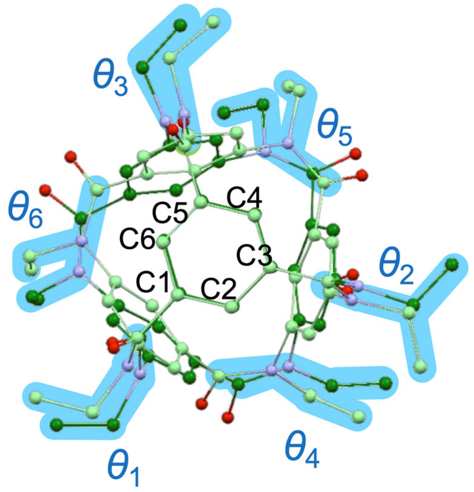

Figure S3. Superimposed structures of (+)-**1a** (R = Et, CCDC code: LOJMIH). Green: molecule C with benzene rings numbered C1-C6; pale green, molecule D with benzene rings numbered C43-C48.

Table S1. Torsion angles in the crystals. Molecule: A and B, R = benzyl ((+)-**1b**, 2419806); C and D, R = Et ((+)-**1a**, CCDC code LOJMIH).

molecule A					Torsion [°]	molecule B					Torsion [°]
θ_1	C25	N1	C26	C27	94.4(5)	θ_1	C141	N103	C142	C143	89.8(5)
θ_2	C33	N2	C34	C35	-100.4(5)	θ_2	C125	N101	C126	C127	-128.7(5)
θ_3	C41	N3	C42	C43	104.1(5)	θ_3	C133	N102	C134	C135	103.7(5)
θ_4	C65	N4	C66	C67	-96.1(5)	θ_4	C149	N104	C150	C151	-112.6(6)
θ_5	C49	N5	C50	C51	-102.2(4)	θ_5	C165	N106	C166	C167	-73.7(6)
θ_6	C57	N6	C58	C59	124.3(4)	θ_6	C157	N105	C158	C159	97.7(5)

molecule C					Torsion [°]	molecule D					Torsion [°]
θ_1	C7	N1	C8	C9	92.2(5)	θ_1	C52	N8	C53	C54	86.4(5)
θ_2	C10	N2	C11	C12	-113.6(5)	θ_2	C55	N9	C56	C57	126.0(5)
θ_3	C13	N3	C14	C15	100.0(5)	θ_3	C49	N7	C50	C51	121.9(5)
θ_4	C22	N4	C23	C24	-156.7(5)	θ_4	C73	N11	C74	C75	-142.9(4)
θ_5	C31	N5	C32	C33	128.1(4)	θ_5	C82	N12	C83	C84	78.5(7)
θ_6	C40	N6	C41	C42	83.8(5)	θ_6	C64	N10	C65	C66	84.5(5)

S5. References

S1. G. M. Sheldrick, SHELXT – Integrated space-group and crystal-structure determination. *Acta Crystallogr.*, 2014,

A70, C1437.

S2. G. M. Sheldrick, Crystal structure refinement with SHELXL. *Acta Crystallogr.*, 2015, **C71**, 3–8.
